# Chemical toxicity prediction based on semi-supervised learning and graph convolutional neural network

**DOI:** 10.1186/s13321-021-00570-8

**Published:** 2021-11-27

**Authors:** Jiarui Chen, Yain-Whar Si, Chon-Wai Un, Shirley W. I. Siu

**Affiliations:** 1 Department of Computer and Information Science, University of Macau, Avenida da Universidade, Taipa, 999078 Macau China; 2 Present Address: Institute of Science and Environment, University of Saint Joseph, Rua de Londres 106, 999078 Macau, China; 3 School of Pharmaceutical Sciences, Universiti Sains Malaysia, USM, 11800 Penang, Malaysia

**Keywords:** Chemical toxicity, Deep learning, Graph convolutional neural network, Semi-supervised learning, Mean teacher, Tox21, ADMET

## Abstract

As safety is one of the most important properties of drugs, chemical toxicology prediction has received increasing attentions in the drug discovery research. Traditionally, researchers rely on in vitro and in vivo experiments to test the toxicity of chemical compounds. However, not only are these experiments time consuming and costly, but experiments that involve animal testing are increasingly subject to ethical concerns. While traditional machine learning (ML) methods have been used in the field with some success, the limited availability of annotated toxicity data is the major hurdle for further improving model performance. Inspired by the success of semi-supervised learning (SSL) algorithms, we propose a Graph Convolution Neural Network (GCN) to predict chemical toxicity and trained the network by the Mean Teacher (MT) SSL algorithm. Using the Tox21 data, our optimal SSL-GCN models for predicting the twelve toxicological endpoints achieve an average ROC-AUC score of 0.757 in the test set, which is a 6% improvement over GCN models trained by supervised learning and conventional ML methods. Our SSL-GCN models also exhibit superior performance when compared to models constructed using the built-in DeepChem ML methods. This study demonstrates that SSL can increase the prediction power of models by learning from unannotated data. The optimal unannotated to annotated data ratio ranges between 1:1 and 4:1. This study demonstrates the success of SSL in chemical toxicity prediction; the same technique is expected to be beneficial to other chemical property prediction tasks by utilizing existing large chemical databases. Our optimal model SSL-GCN is hosted on an online server accessible through: https://app.cbbio.online/ssl-gcn/home.

**Supplementary information:**

**Supplementary information** accompanies this paper at 10.1186/s13321-021-00570-8.

## Introduction

The fundamental strategy in modern drug discovery and development is to identify chemical compounds that potently and selectively modulate the functions of the target molecules to elicit a desired biological response. How to quickly locate these compounds from the vast chemical space and then determine their drug-like properties remains a major challenge [1–3]. Traditionally, chemists and biologists perform in vitro and in vivo experiments to test the pharmacodynamics and pharmacokinetic (PD/PK) properties of selected candidates obtained from initial screening results [4, 5]. However, these experiments are not only very costly in terms of time and money, the experiments that involve animal testings are increasingly questionable from ethical perspectives [6]. Previous studies show that it typically takes 6 to 12 years and more than 2.6 billion dollars to develop a new drug. Of this cost, about 1.1 billion dollars is for the drug development phases prior to human testing [7].

Toxicity is one of the five pharmacokinetic properties (ADMET) that must be strictly ascertained before a new drug candidate is approved for clinical trials [8]. On the premise that “*the structure of a chemical substance implicitly determines its physical and chemical properties and reactivity, and these properties interact with biological systems to determine its biological/toxicological properties*” [9, 10], efforts have been made to develop computational methods, often machine learning (ML) based, that attempt to relate the toxicological properties of compounds to their chemical structures. For a comprehensive review of ML-based toxicity prediction methods, the readers are referred to refs [11–13].

Graph Convolutional Neural Networks (GCN) are commonly used for tasks such as social network analysis and knowledge graph mining. Since biomolecular structures can also be represented as graphs, a variety of GCN-based biomolecular property prediction models have been developed in recent years. For example, the Weave model was proposed by Kearnes et al. in 2016 [14], which was a deep learning system based on molecular graph convolutions. This model uses only the simple descriptions of atoms, bonds, and atom pairs as input data. In addition, a learnable module called Weave module, extracts and combines the features of atom and distance relationship with learnable parameters. These modules can be stacked to an arbitrary depth to allow fine-tuning of the architecture for the needs of different learning tasks. In 2017, Li et al. proposed the GraphConv-SuperNode model [15]. By adding a dummy fully connected node (the super node) in each graph, this model captures and extracts graph-level representations from chemical structures, allowing it to focus on graph-level classification and regression tasks. In 2020, Wang et al. proposed a graph attention convolutional neural network (GACNN) that classified poisonous chemicals to honey bees [16], which is a Graph Convolution Neural Network with undirected graph and attention mechanism. They demonstrated that the performance of their GACNN model was better than all previous models, and they also summarised important structural features that might lead to poisoning.

All of these previous studies have highlighted the advantages of using GCN-based models to predict biomolecular properties. First, the suitability of different traditional molecular descriptors for different tasks significantly affects the performance of the models [16, 17]. Graph-based molecular representations can circumvent this problem by preserving the structural and physicochemical information of the molecules. Second, the majority of models using graph-based techniques perform better on biomolecular property prediction tasks than conventional ML models using traditional molecular descriptors [14–16, 18]. Third, since GCN-based models can directly manipulate graph-based molecular representations, they can retain molecular structural information during prediction. This characteristics of GCN makes the interpretability of GCN-based models superior to other traditional ML models.

Based on the different training strategies, ML algorithms can be broadly classified into 4 types, namely supervised learning (SL), semi-supervised learning (SSL), unsupervised learning and reinforcement learning [19]. All the prediction models we mentioned above are based on the SL algorithms which learn only from annotated datasets. However, despite enormous efforts in data curation and data sharing, the amount of labeled data falls far short of the amount of known compounds. Strategies to make use of the unannotated data such as those of SSL are expected to enhance the generalizability of prediction models.

Therefore, inspired by the success of GCN and the needs for improving chemical toxicity prediction confronted with limited data, we designed a learning system that hybridizes graph convolutional neural network (GCN) and SSL to predict the toxicity of chemical compounds. Here, we used chemical data from the Tox21 dataset as annotated data and collected compounds from other datasets as unannotated data. First, the molecular features encoded in GCN were defined, then experiments were performed to investigate the influence of SSL on the predictivity of the models. Moreover, the performances of the SSL models with varying unannotated data ratios were compared, which showed that SSL has a positive influence on the prediction performance of GCN models.

This paper is organized as follows. The theoretical foundation of GCN and the mean teacher SSL algorithm are presented in the Material and Method section. The dataset, model, and validation technique are then described. The Results section contains comparative study of the traditional ML, SL-GCN, and SSL-GCN models performances. The impact of various unannotated data ratios was also investigated. Finally, SSL-GCN was compared to existing DeepChem methods for toxicity prediction.

## Material and method

### Graph convolutional neural network (GCN)

Traditional convolutional neural networks (CNN) can extract features from Euclidean or grid structure data, such as images and text. But for non-Euclidean data like social networks, knowledge graphs, or chemical structures, due to its irregular data topology, CNN cannot directly operate on them [20, 21]. A solution for machine learning on non-Euclidean data is Graph Convolutional Neural Network (GCN) [22]. GCN has been widely used in solving computer science problems such as social network analysis [23], natural language processing [24, 25], and recommendation system [26, 27], and also chemistry problems such as molecular properties prediction [14, 15, 18, 20]. For the latter, each molecule is described as an undirected graph where atoms are represented as nodes and covalent chemical bonds are represented as edges. The basic idea of graph convolution is to apply a learnable function on each node and its neighbors, gradually merging information from distant atoms through the connecting edges, and ultimately extracting the atom-type and connectivity patterns in the molecule. In this work, we used off-the-shelf GCN method that was proposed by Kipf et al. in 2017 [28]. The layer-wise propagation function of this approach is defined in the following equations in terms of matrix calculation:

1 $$\begin{aligned}&{\tilde{A}} = A + I \end{aligned}$$

2 $$\begin{aligned}&H^{(l+1)}=\sigma ({\tilde{D}}^{-\frac{1}{2}}{\tilde{A}}{\tilde{D}}^{-\frac{1}{2}}H^{(l)}W^{(l)}) \end{aligned}$$

These equations can be denoted as $f(H^{(l)}, A)$. ${\tilde{A}}$ represents the adjacency matrix *A* of an undirected graph ${\mathcal {G}}$ with added self-connections *I*. ${\tilde{D}}$ is the degree matrix of ${\tilde{A}}$. $H^{(l)}\in {\mathbb {R}}^{N\times D}$ represents the nodes signal matrix (features) generated by the $l{\rm th}$ layer, where *N* and *D* denote the number of nodes in this graph and the dimension of each node’s signal matrix respectively. $W^{(l)}$ is the layer-specific learnable weight matrix of the $l^{th}$ layer. $\sigma$ denotes a non-linear activation function [28].

To facilitate implementation, the previous equations can be represented as the following:

3 $$\begin{aligned} h_{i}^{(l+1)} = ReLU\left( b^{(l)}+\sum _{j\in {\mathcal {N}}(i)}\frac{1}{\sqrt{\left| {\mathcal {N}}(i) \right| } \sqrt{\left| {\mathcal {N}}(j) \right| }}h_{j}^{(l)}W^{(l)} \right) \end{aligned}$$

where ${\mathcal {N}}(i)$ is the set of neighbors of the node *i*. $W^{(l)}$ represents the layer-specific learnable weight matrix of the $l{th}$ layer, $h_{j}^{(l)}$ is the signal matrix (features) of each neighbor node *j* around *i*, and $b^{(l)}$ is the bias value of the $l^{th}$ layer. Therefore, the signal of each node in the next layer is determined by the weighted sum of signals in each node of the current layer and the signals of its adjacent nodes of the same layer. All signals are nonlinearly transformed using the Rectified Linear Unit (ReLU) function, $ReLU(x)=max(0,x)$.

### Semi-supervised learning (SSL)

The basic idea of machine learning (ML) is to reproduce the human learning process by computer algorithms. Most ML algorithms can be classified into four types [19, 29]: supervised learning, unsupervised learning, semi-supervised learning and reinforcement learning. The most commonly used method is supervised learning. It derives knowledge from training data with fully annotated labels [30]. However, acquiring accurate annotated data is sometimes difficult for certain tasks such as chemical compound properties prediction. On one hand, there are tens of thousands known chemical compounds that exist in nature, and even more artificial chemical compounds are being produced every year. On the other hand, each annotation requires labor-intensive and expensive procedure from compound synthesis to measurement. Consequently, a significant amount of molecules are not properly labelled while some labels may subject to experimental errors. To learn from incompletely annotated data, semi-supervised learning method is more suitable [31].

In SSL, it is assumed that the label function is smooth in high-density areas, so data points located in the same area should share the same label. Based on this smoothness assumption, even unlabelled data can be exploited in the learning process. Here, the main idea is to build classification models that are robust to local perturbations in the input data. When the input data is perturbed with a small amount of noise, the prediction results for the perturbed data and original data should be similar [32]. Since this consistency in predictions does not depend on the data labels, therefore unlabelled data can be exploited in the training process to enhance the prediction consistency of the model.

Earlier SSL models that used this consistency regularization, such as the $\Gamma$-model [33], assigned two roles (teacher and student) to the same model. With the role of student, the model learns based on labeled data. With the teacher role, the model generates targets for unlabeled data, which are then used by itself as a student for consistency learning. However, at the beginning of training, the generated targets for unlabeled data are most likely incorrect. The consistency cost for unlabeled data outweighs the classification cost for labeled data at the beginning of training, so the model cannot learn any new information from the training process [34]. One way to solve this problem is to carefully select or update the teacher model instead of sharing the same model with the student model. Following this idea, the $\Pi$-model and Temporal Ensembling model were proposed in 2017 [35].

In each training epoch of the $\Pi$-model, the same unlabeled data are predicted twice with different roles (student and teacher). Since data perturbations and dropout methods are implemented in each prediction process, two prediction processes will give slightly different predictions for the same data. The goal of the $\Pi$-model during the training process is to make two predictions for the same unlabeled data as consistent as possible. Their experiments show that this method can eventually make the teacher model make accurate targets for unlabeled data [35]. However, the computational cost of this model is too high. The Temporal Ensembling model improves on the $\Pi$-model by making predictions only once per training epoch for unlabeled data, reducing the number of predictions by half and nearly doubling the speed. To calculate the consistency cost in the Temporal Ensembling model, the target of unlabeled data is generated by the exponential moving average (EMA) of the predictions for unlabeled data in previous training epochs. However, since each target is updated only once per epoch, the updating speed is too slow, which still limits the training speed of Temporal Ensembling model [34].

In this study, we implemented the SSL algorithm proposed by Tarvainen and Valpola, called Mean Teacher (MT) [34]. To circumvent the limitations of the Temporal Ensembling model, the MT algorithm updates the internal weights of the model through the EMA strategy at each training step to produce a more accurate model, rather than updating the targets of the unlabeled data at each training epoch. During training process, this algorithm requires two models with the same architecture, namely the student model and the teacher model. In each training step, the student model updates its internal weights based on the classification loss on the labeled data and the consistency loss between the two models on the unlabeled data. After the student model is updated, the teacher model is also updated using EMA strategy defined in Equation 4 [31, 34]. Previous studies have demonstrated that this kind of self-ensembling framework could bring improvements to classification models [34, 35]. The pseudo code of this algorithm is shown below:

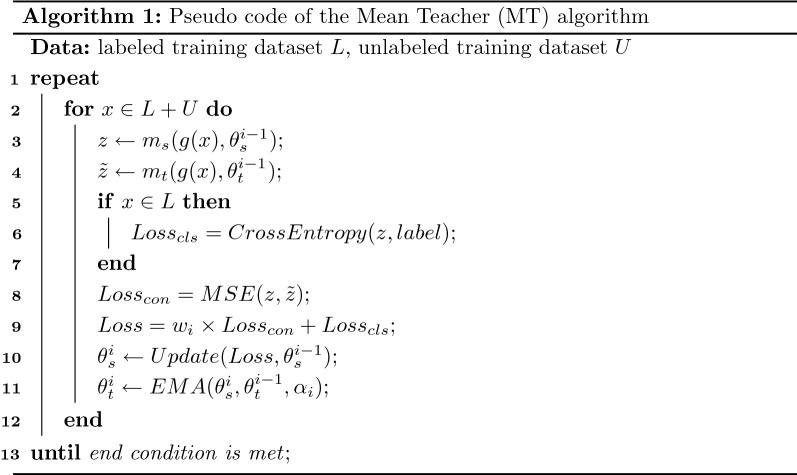



$g(\cdot )$ denotes the data perturbation function, $m_{s}(\cdot )$ and $m_{t}(\cdot )$ represent the student and teacher models respectively, $\theta _s^i$ and $\theta _t^i$ represent the internal weights in the training step *i*, *z* and ${\tilde{z}}$ are the generated classification probabilities. $Loss_{cls}$ and $Loss_{con}$ represent classification loss and consistency loss. $w_{i}$ denotes the consistency loss coefficient in the training step *i*. This consistency loss coefficient varies with the training steps. It is defined as the function $e^{-5(1-t)^2}$, where $t\in \{0,1\}$, represents scaled number of training step [34]. $Update(\cdot )$ is the process of updating the internal weights of the model through backpropagation.

$EMA(\cdot )$ is the process of updating the weights in $m_{t}$ by applying the Exponential Moving Average (EMA) of weights in $m_{s}$ where $\alpha _{i}$ is the smoothing coefficient. The following equation defines this process mathematically:

4 $$\begin{aligned} \theta _{t}^{i} = \alpha _{i}\theta _{t}^{i-1} +(1-\alpha _{i})\theta _{s}^{i} \end{aligned}$$

In our implementation, we applied the Gaussian noise *g*(*x*) as the data perturbation method using the same distribution for both $m_{s}(\cdot )$ and $m_{t}(\cdot )$. The cross entropy loss function and Mean Squared Error (MSE) are used to compute the classification loss and consistency loss, respectively. The GCN network is optimized using the Adam optimizer [36], which is the optimizer chosen in the original implementation of MT [34]. Although both the well-trained teacher model and the student model can be used for prediction, previous studies have demonstrated that the teacher model is more accurate than the student model [31, 34]. Therefore, the teacher model is used as the final classification model.

### Datasets

For semi-supervised learning, both labeled (compounds with toxicity information) and unlabeled (compounds without toxicity information) data are required. In this study, the Tox21 dataset from MoleculeNet [37] is used as the labeled data. The Tox21 challenge is a community-wide compound toxicity prediction competition in 2014. Since then, the Tox21 dataset has been widely used as the benchmark dataset for evaluating toxicity prediction models. It consists of 12 endpoints, including 7 nuclear receptor signals (NR-AR, NR-AhR, NR-AR-LBD, NR-ER, NR-ER-LBD, NR-Aromatase, NR-PPAR-gamma) and 5 stress response indicators (SR-ARE, SR-ATAD5, SR-HSE, SR-MMP, SR-p53). In this dataset, each compound is expressed in Simplified Molecular Input Line Entry Specification (SMILES) format and the binary labels indicate whether the compound is toxic to a specific toxicological endpoint. In total, the Tox21 dataset include 7831 compounds and 12 different endpoints. It should be noted that not all compounds have all endpoint labels; the missing endpoint label means that the toxicology effect toward this endpoint is unknown. For unlabeled data, other chemical compound datasets were sought from the MoleculeNet website, including ClinTox, SIDER, ToxCast, and HIV datasets [37]. All the label information in these datasets have been removed. In addition, duplicate molecules between these datasets and the Tox21 dataset have also been removed. In total, 50527 compounds were used as unlabeled data. Table 1 shows the details of the datasets used in this study.

**Table 1:** The labeled compound toxicity datasets for 12 toxicological endpoints and the unlabeled dataset

Endpoint	Compounds(labeled)	Training set	Validation set	Test set
NR-AhR	6549	5239	655	655
NR-AR-LBD	6758	5406	676	676
NR-AR	7265	5812	726	727
NR-Aromatase	5821	4656	582	583
NR-ER-LBD	6955	5564	695	696
NR-ER	6193	4954	619	620
NR-PPAR-gamma	6450	5160	645	645
SR-ARE	5832	4665	583	584
SR-ATAD5	7072	5657	707	708
SR-HSE	6467	5173	647	647
SR-MMP	5810	4648	581	581
SR-p53	6774	5419	677	678
Unlabeled data	50527	–	–	–

For each labeled dataset, we follow the conventional dataset splitting rule with the splitting ratios of 0.8:0.1:0.1 to divide the dataset into training, validation and test sets. Training set is used for the training process, validation set for the hyperparameter tuning process and the test set is to measure the generalization performance. The most commonly used splitting method is random splitting. However, it is not always suitable for molecular data because random splitting cannot guarantee that the training and test sets contain diverse and representative data samples [37, 38]. In order to overcome the problem of data bias, we adopted a scaffold splitting method. It splits the dataset according to the two-dimensional structural framework of the molecule [39, 40] and then assign structurally different molecules into different subsets [37]. In this way, both the training set and the test set contain a good proportion of data samples scattered in the molecular space of the dataset, and we can expect that the performance of the model measured on this test set is closer to its actual performance on new data.

**Fig. 1:**
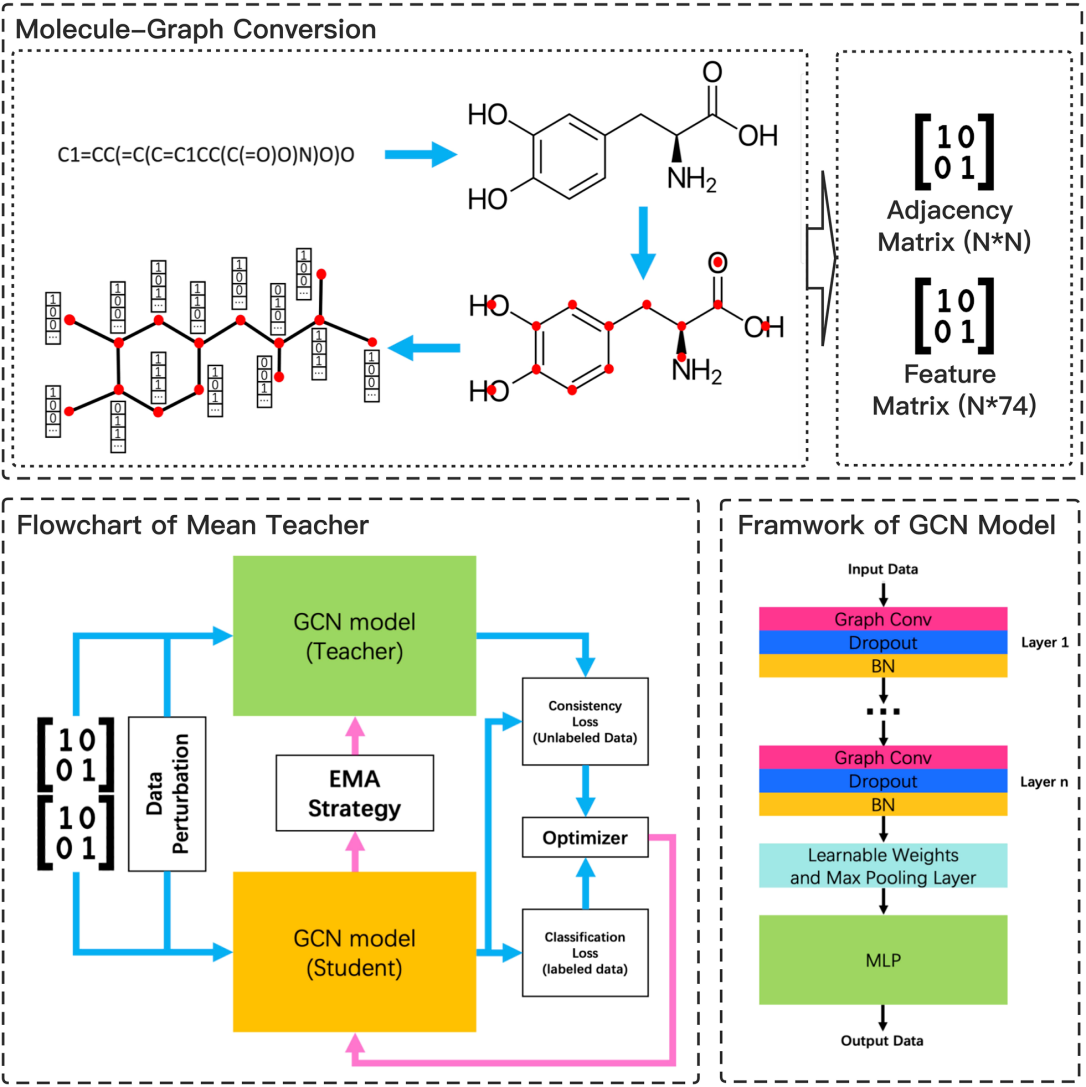
The SSL-GCN model for compound toxicity prediction. Molecular compounds are converted into graphs of nodes and connections. The GCN model architecture is composed of two stacked layers of graph convolutional layer, dropout, and batch normalization layer. All signals are summarized by the max pooling layer and fed into the multilayer perceptron network to generate the final output. The teacher and student GCN models are updated using the MT algorithm

As mentioned above, an undirected graph can be described by two matrices, namely the signal (feature) matrix *H* and the adjacency matrix *A*. In this study, we used the molecule-graph conversion tool from Deep Graph Library (DGL) [41] to convert molecules from SMILES to graphs. For each molecule, the connectivity of atoms is stored in the adjacency matrix and the physicochemical properties of each atom (node features) are encoded into a feature matrix in binary or numerical form. Since the DGL conversion tool provides eight default atom features, as listed in Table 2, the dimension of each node feature matrix is $1\times 74$. Therefore, for a molecule with *N* atoms, the conversion will generate one adjacency matrix of dimension $N\times N$ and one feature matrix of dimension $N\times 74$. This graph conversion process is depicted in Fig. 1. After this step, the graph-based molecular data can be learned by the graph convolutional neural network.

**Table 2:** Atom features provided by the molecule-graph conversion tool from Deep Graph Library

No.	Description	No. of bits	Form
1	One hot encoding of the atom type	1-43	Binary
2	One hot encoding of the atom degree	44-54	Binary
3	One hot encoding of the number of implicit Hs on the atom	55-61	Binary
4	Formal charge of the atom	62	Numerical
5	Number of radical electrons of the atom	63	Numerical
6	One hot encoding of the atom hybridization	64-68	Binary
7	Whether the atom is aromatic	69	Numerical
8	One hot encoding of the number of total Hs on the atom	70-74	Binary

### Model architecture and hyperparameters selection

The architecture of our GCN model consists of two parts, an encoder and a classifier. The encoder extracts and updates node representations through several graph convolutional layers (Graph Conv). In addition, there is a dropout layer after each Graph Conv layer to provide additional noise to the molecular representations [31, 34]. The last layer of the encoder merges all nodes features into a tensor by using max-pooling and weighted sum operations. This tensor is the learned representation of the input molecule. The classifier is to compute the final prediction. We used the classifier provided in DGL [41] which contains two layers perceptron (MLP) with a dropout layer and a batch normalization layer.

In order to select the best hyperparameters for these models, Bayesian optimization algorithm [42] is used to search the hyperparameter space, and the maximum number of trials is 32. In each trial, the algorithm selects a set of candidate hyperparameters and initializes the model. Then, model training and validation are carried out iteratively until the early stopping condition of 30 epochs is met. After all trials are completed, a set of candidate hyperparameters with the best validation metric (ROC-AUC) is selected as the default hyperparameters for the following experiments.

Since the toxicity dataset is highly imbalanced, with an average toxic/non-toxic data ratio of about 1:17, the area under the Receiver Characteristic Operator curve (ROC-AUC) is used as the main metric in the hyperparameter selection process (practically, to decide for early stopping) and the final model evaluation. The hyperparameters with the best validation performance are selected to construct the optimal toxicity prediction models. Finally, the generalization performance of these models are estimated using the test set.

### Implementation detail

In this study, all implementations and experiments are carried out in an environment with following libraries/software: Python 3.7.9, Anaconda 4.7.10, Scikit-learn 0.23.2, RDKit v2018.09.3.0. We used Pytorch 1.7.0 with CUDA 10.0 as the basic machine learning framework. The GCN model is implemented using DGL 0.5.6 and its supplementary package DGL-LifeSci 0.2.6 [41] (available on GitHub, DGL [43], DGL-LifeSci [44]). The Bayesian Optimization process for hyperparameter selection is implemented using Hyperopt 0.2.5 [42] (available on GitHub [45]). We also used DeepChem 2.5.0 [46] to generate the benchmark scores of other state-of-the-art models on the Tox21 dataset (available on GitHub [47]). The original source code for the Mean Teacher(MT) algorithm [34] can be accessed via its GitHub repository [48].

## Results

All experiments were repeated five times to observe the variability of the results and obtain an accurate measure of model performance through the average ROC-AUC score. The complete record of all experiments can be found in the Additional file 1.

### Performance of conventional machine learning (ML) methods

To establish the baseline performance, several commonly used ML algorithms, namely K-Nearest Neighbor (KNN), Neural Network (NN), Random Forest (RF), Support Vector Machine (SVM) and eXtreme Gradient Boosting (XGBoost) were tested. The compounds were encoded using the Extended Connectivity Fingerprints (ECFP4), which is a circular topological fingerprint designed for molecular characterization, similarity searching, and structure-activity modeling [49]. The encoding was generated using the RDKit library. In total, 60 different ML models (12 prediction tasks $\times$ 5 types of ML algorithms) were trained and optimized using the training and validation sets. Subsequently, the optimal models were tested on the test set. The test performance of these conventional models on the 12 toxicity prediction tasks are presented in Table 3. Each experiment was repeated 5 times; the average ROC-AUC score and the standard deviation (std) were reported. In all prediction tasks, the ROC-AUC scores range between 0.5127 and 0.8287. In certain cases (KNN, SVM, and XGBoost), we observed that the same optimal models were obtained in all replicate experiments such that the ROC-AUC scores are the same (std = 0). Overall, RF, XGBoost, and SVM generated the best models for 5, 4, 3 of the prediction tasks, respectively. The average ROC-AUC score of the best performing conventional ML models of all tasks is 0.71.

**Table 3:** The average test performance of conventional ML models on the 12 prediction tasks in 5 repeated experiments

Tasks	KNN		NN		RF		SVM		XGBoost	
	AUC	Std.	AUC	Std.	AUC	Std.	AUC	Std.	AUC	Std.
NR-AR-LBD	0.6955	–	0.6671	0.0244	**0.7323**	0.0267	0.6795	–	0.6784	–
NR-AR	0.6527	–	0.6806	0.0088	0.6836	0.0266	**0.7193**	–	0.6818	–
NR-AhR	0.7639	–	0.7628	0.0177	0.8243	0.0074	0.7794	–	**0.8287**	–
NR-Aromatase	0.5576	–	0.5127	0.0772	0.6900	0.0092	0.6873	–	**0.7106**	–
NR-ER-LBD	0.6191	–	0.5387	0.1171	0.6169	0.0300	0.6078	–	**0.6250**	–
NR-ER	0.6597	–	0.6549	0.0162	0.6316	0.0080	0.6126	–	**0.6745**	–
NR-PPAR-gamma	0.6182	–	0.5558	0.0736	**0.7135**	0.0258	0.6454	–	0.6414	–
SR-ARE	0.6366	–	0.5656	0.0251	0.6603	0.0018	**0.6843**	–	0.6640	–
SR-ATAD5	0.5866	–	0.6240	0.0537	**0.6928**	0.0189	0.6546	–	0.6841	–
SR-HSE	0.6574	–	0.6143	0.0222	0.6852	0.0131	**0.6858**	–	0.6647	–
SR-MMP	0.7057	–	0.6551	0.0612	**0.7818**	0.0065	0.7794	–	0.7656	–
SR-p53	0.6778	–	0.5963	0.0075	**0.7263**	0.0130	0.7051	–	0.6942	–

The bold number denotes the best result among all conventional ML models in the corresponding task

### Performance of supervised learning GCN (SL-GCN)

**Fig. 2:**
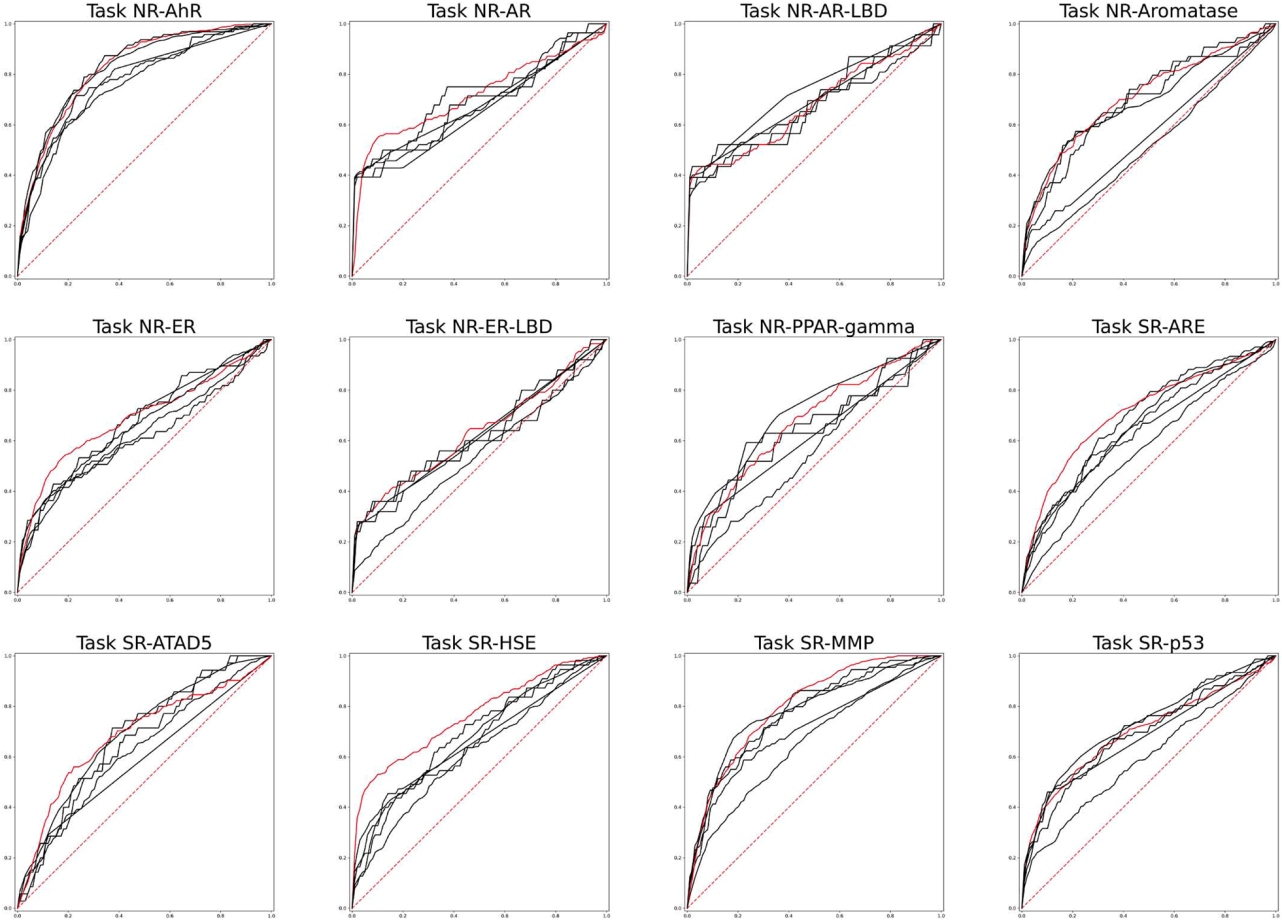
ROC curves of conventional ML models and SL-GCN models. The comparison of ROC curves between conventional ML models (black line) and SL-GCN models (red line) on 12 toxicity prediction tasks. Additional information of the ROC curves are provided in the Additional file 1

Having established the baseline performance of the traditional ML models in toxicity prediction, we went on to test the GCN models for the 12 prediction tasks. Similar to other ML models above, the GCN models were trained using supervised learning and optimized by the Bayesian optimization algorithm, hence the name SL-GCN. In Fig. 2, the ROC curves of the SL-GCN models on the test set prediction are plotted against other ML models, and the 5-repeated average of the ROC-AUC scores are tabulated in Table 4. The results show that, while the SL-GCN models perform similarly to the best conventional ML models in the majority of the twelve toxicity prediction tasks, they improve in four of the tasks, including NR-ER, SR-ARE, SR-HSE, and SR-MMP, while they perform worse in three of the tasks, including NR-AR-LBD, NR-PPAR-gamma, SR-p53.

**Table 4:** The average test performance of SSL-GCN models with various unlabeled data ratio ($R_{u}$ in brackets) on the 12 prediction tasks in 5 repeated experiments. For comparison, the results of the SL-GCN models are shown

Tasks	SL-GCN		SSL-GCN (0.5)		SSL-GCN (1.0)	
	AUC	Std.	AUC	Std.	AUC	Std.
NR-AR-LBD	0.6783	0.0269	0.7417	0.0105	0.7333	0.0401
NR-AR	0.7157	0.0367	0.7550	0.0483	0.7858	0.0357
NR-AhR	0.8260	0.0055	0.8161	0.0121	0.8295	0.0129
NR-Aromatase	0.7092	0.0167	0.7202	0.0057	0.7306	0.0156
NR-ER-LBD	0.6340	0.0161	0.6623	0.0330	0.6794	0.0411
NR-ER	0.6899	0.0160	**0.7188**	0.0196	0.7114	0.0179
NR-PPAR-gamma	0.6753	0.0278	0.7267	0.0210	**0.7614**	0.0212
SR-ARE	0.7134	0.0137	0.7241	0.0065	0.7288	0.0063
SR-ATAD5	0.6850	0.0223	0.7119	0.0080	0.7061	0.0245
SR-HSE	0.7644	0.0096	0.7636	0.0239	0.7678	0.0080
SR-MMP	0.7988	0.0066	**0.8120**	0.0075	0.8035	0.0061
SR-p53	0.6970	0.0253	0.7291	0.0114	0.7401	0.0203

Tasks	SSL-GCN (2.0)		SSL-GCN (3.0)		SSL-GCN (4.0)	
	AUC	Std.	AUC	Std.	AUC	Std.
NR-AR-LBD	**0.7647**	0.0279	0.7377	0.0145	0.7477	0.0135
NR-AR	0.7512	0.0358	0.7412	0.0659	**0.7967**	0.0251
NR-AhR	0.8287	0.0072	**0.8303**	0.0055	0.8224	0.0090
NR-Aromatase	0.7232	0.0040	0.7287	0.0082	**0.7337**	0.0057
NR-ER-LBD	0.6772	0.0161	0.6662	0.0250	**0.6870**	0.0282
NR-ER	0.7039	0.0124	0.7113	0.0083	0.7166	0.0137
NR-PPAR-gamma	0.7491	0.0201	0.7429	0.0177	0.7456	0.0223
SR-ARE	**0.7297**	0.0080	0.7277	0.0067	0.7243	0.0114
SR-ATAD5	0.7096	0.0139	**0.7175**	0.0143	0.7077	0.0162
SR-HSE	**0.7822**	0.0097	0.7731	0.0098	0.7700	0.0066
SR-MMP	0.8100	0.0033	0.8031	0.0088	0.8081	0.0078
SR-p53	**0.7518**	0.0198	0.7359	0.0147	0.7434	0.0126

The bold number denotes the best result among all SSL-GCN models with various unlabeled data ratio in the corresponding task

### Performance of semi-supervised learning GCN (SSL-GCN)

**Fig. 3:**
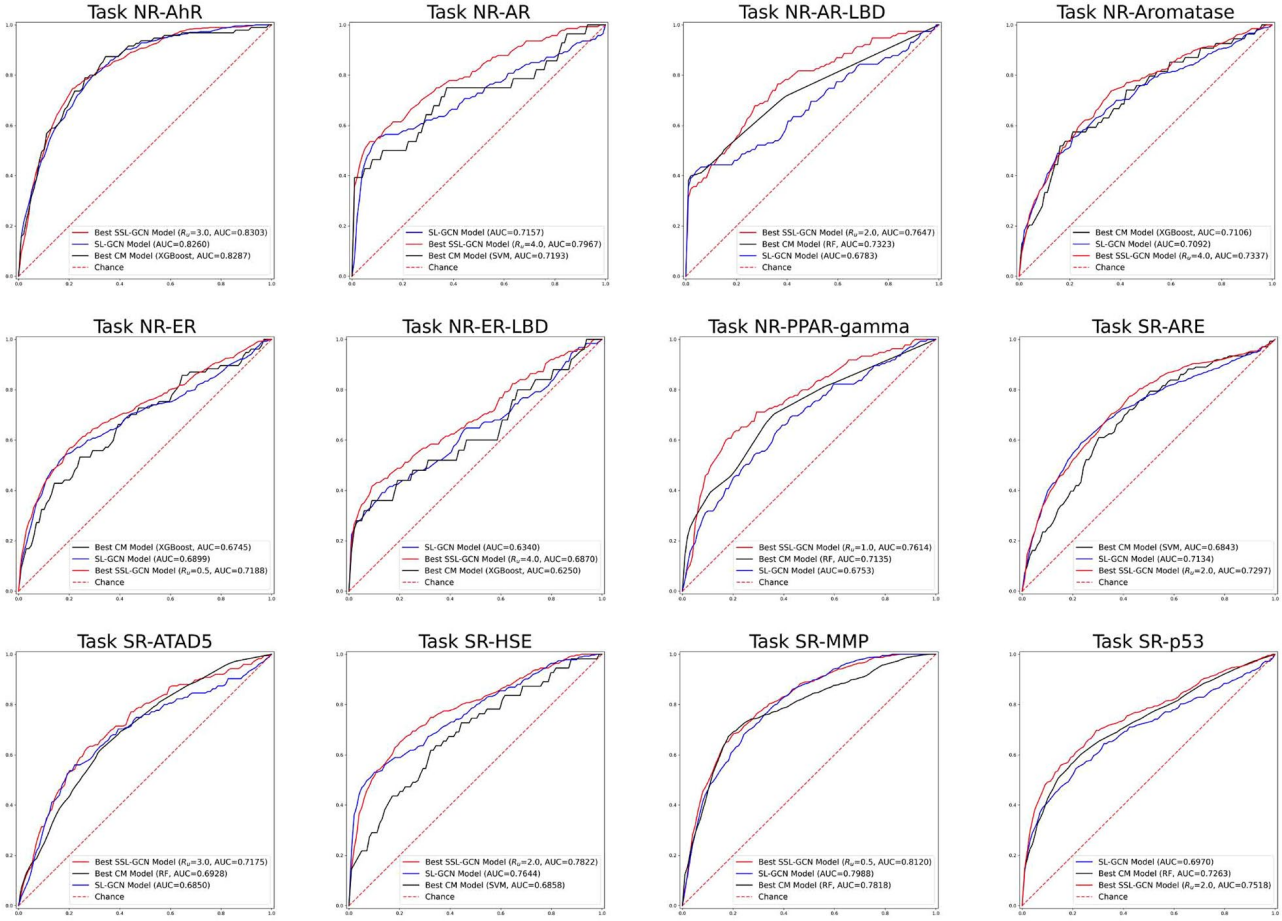
ROC curves of best SSL-GCN, SL-GCN, and CM models. The comparison of ROC curves between the best conventional ML models (CM, black line), SL-GCN models (blue line), and SSL-GCN models with the best $R_{u}$ (red line) on 12 toxicity prediction tasks. Additional information on the ROC curves can be found in the Additional file 1

The MT technique employed in this study necessitates the use of two models with the same architecture, one for $m_t$ and one for $m_s$. Therefore, we used the hyperparameters obtained from the SL-GCN models as the initial parameters to train SSL-GCN. As shown in the previous study [34], the amount of unlabeled data in the training process can affect the final model performance. To investigate this impact on the performance of the SSL-GCN models, we ran numerous trials with varying amounts of unlabeled data. We define the unlabeled-to-labeled data ratio as $R_u \in \{0.5, 1.0, 2.0, 3.0, 4.0\}$. So, when $R_u=0.5$, we randomly select a portion of unlabeled data from the entire unlabeled data set to participate in the semi-supervised learning process, and the amount of this portion of unlabeled data is only half of the labeled data. Due to significant increase in training time, a large $R_u$, such as $> 4.0$, were not considered. Table 4 shows the test results of the optimized SSL-GCN models for the 12 toxicity prediction tasks, as well as a comparison of the ROC curves in Fig. 3.

**Fig. 4:**
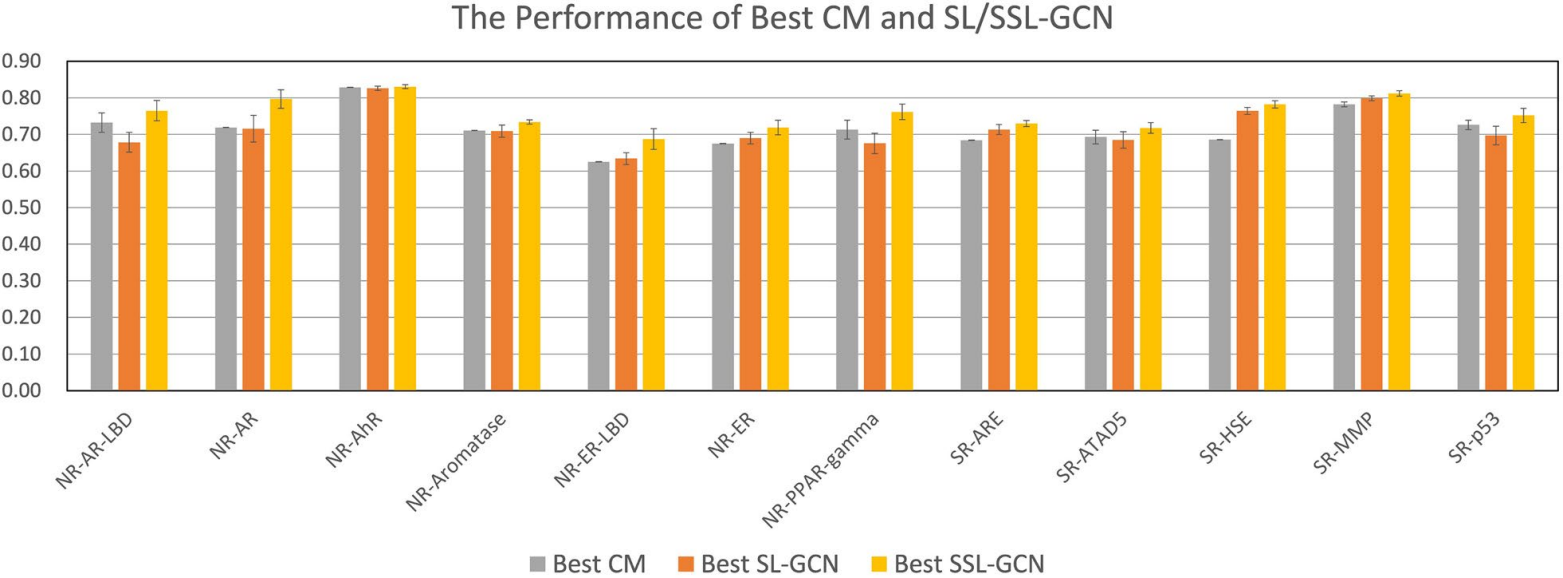
Comparison of AUC scores between SL-GCN, SSL-GCN and CM models Comparison of the best models from conventional methods (CM), SL-GCN, and the SSL-GCN on twelve toxicity prediction tasks. The mean and standard deviation are obtained from the 5-repeat experiments

As shown in Table 4, SSL improves the predictive power of the GCN models when sufficient amount of unlabeled data is included in the training. SSL-GCN with $R_u$ of 0.5 improves the ROC-AUC score in 10 of the 12 prediction tasks, while only the ROC-AUC scores of two tasks are somewhat reduced. When the SSL-GCN models are trained with additional unlabeled data ($R_u$= 1.0 to 4.0) , they always outperform their SL-GCN counterparts in terms of AUC score. Nonetheless, the best $R_u$ for each prediction task is different. SSL-GCN produces 4 optimal models when $R_u=2.0$; 3 optimal models when $R_u=4.0$; 2 optimal models when $R_u=0.5$, and 1 optimal model when $R_u=1.0$. As a result, the best $R_u$ varies depending on the prediction task at hand. The rates of performance improvement in terms of ROC-AUC for different task range from 1% to 13%. Finally, Fig. 4 compares the best CM, SL-GCN and SSL-GCN models. As can be clearly seen, SSL-GCN can produce models with greater predictive potential than CM and SL-GCN in all toxicity prediction tasks.

As a summary, the comparative study of the SSL-GCN models with varying $R_u$ values suggests that when training with unlabeled data, the ratio of unlabeled and labeled data should be treated as a hyperparameter in order to obtain the optimal model.

### Case study: how the similarity between unlabeled and labeled data affects the semi-supervised learning process?

In the previous section, we showed that semi-supervised learning algorithms can improve the performance of our GCN models compared to models trained with purely supervised algorithm. However, we only studied the effect of unlabeled data ration $R_{u}$ on the SSL algorithm. Here, we will further investigate how the similarity between unlabeled and labeled data affects the performance of SSL-GCN model.

To define the similarity between unlabeled data and labeled dataset, we used the k-nearest neighbors (KNN) method proposed by Tropsha et al. [50, 51] This method are been widely used to measure the similarity between known and unknown chemical compounds using different similarity cutoff, $C_s$, which is defined by following equation 5.

5 $$\begin{aligned} C_s(Z) = <d> + Z\sigma \end{aligned}$$

where $<d>$ denotes the average of similarity scores of all instances in labeled data set, $\sigma$ denotes the standard deviation of these similarity scores. *Z* is a self-defined parameter to control the similarity cutoff $C_s$, which can help us determine the level of similarity. Next, we used the average similarity score $SS_i$ between each unlabeled instance *i* and its k nearest neighbors in the labeled dataset to evaluate how similar each unlabeled instance is to the labeled dataset. In this study, $k=5$ and we used RDKit to calculate the most commonly used Tanimoto (Jaccard) distance as similarity score. To properly define the level of similarity, we first counted the distribution of $SS_i$ in 12 similarity domains defined by different cutoff values $C_s$. The *Z* of these cutoff values range from − 2 to 3.5 with a step size of 0.5. The detail of the distribution can be found in the Additional file 1: Figure S4.

To shorten the experiment time and to ensure that there is enough unlabeled data at each similarity level to support the semi-supervised learning process, we reorganized the above 12 similarity domains into 3 similarity domains based on the distribution, namely close, normal, and far. For one unlabeled instance *i* with similarity score $SS_i$, $SS_i \le C_s(Z=0)$ means *i* belongs to close domain; $C_s(Z=0) < SS_i \le C_s(Z=1)$ means it belongs to normal domain; $C_s(Z=1) < SS_i$ represents *i* belongs to far domain. Based on three similarity domains, we divided the entire unlabeled dataset into three subsets with corresponding similarity level. The following Table 5 presents the detail of these unlabeled subsets.

**Table 5:** The subsets of unlabeled toxicity compounds for 12 toxicological endpoints with varying levels of similarity to the corresponding labeled dataset

Endpoint	Compounds(close)	Compounds(normal)	Compounds(far)	Total
NR-AhR	12116	20416	17995	50527
NR-AR-LBD	11765	20379	18383	50527
NR-AR	12471	19857	18199	50527
NR-Aromatase	11658	20764	18105	50527
NR-ER-LBD	11930	20140	18457	50527
NR-ER	11868	20527	18132	50527
NR-PPAR-gamma	11527	20797	18203	50527
SR-ARE	11659	21301	17567	50527
SR-ATAD5	12309	19875	18343	50527
SR-HSE	12534	20640	17353	50527
SR-MMP	11552	21325	17650	50527
SR-p53	12239	19991	18297	50527

Here, we used these newly generated subsets to train several SSL-GCN models for comparison. We adopted the same experimental procedure (repeated 5 times) and optimal hyperparameter settings as in the previous section to facilitate performance comparison. The average ROC-AUC scores of these SSL-GCN models on the 12 test sets can be found in Table 6. The bold number denotes the best result among all models (all, close, normal, far) in the corresponding task, the underlined number represents only the best result among models using different similarity levels of unlabeled subsets (close, normal, far).

**Table 6:** The average test performance of the SSL-GCN models with different similarity levels of unlabeled subsets (close, normal, far) on the 12 prediction tasks in 5 repeated experiments

Tasks	SSL-GCN (all)		SSL-GCN (close)		SSL-GCN (normal)		SSL-GCN (far)	
	AUC	Std	AUC	Std	AUC	Std	AUC	Std
NR-AR-LBD	0.7647	0.0279	0.7353	0.0353	0.7410	0.0210	**0.7726**	0.0242
NR-AR	**0.7967**	0.0251	0.7398	0.0594	0.7389	0.0401	0.7351	0.0357
NR-AhR	**0.8303**	0.0055	0.8261	0.0076	0.8292	0.0080	0.8278	0.0055
NR-Aromatase	0.7337	0.0057	0.7318	0.0082	0.7222	0.0131	**0.7382**	0.0145
NR-ER-LBD	**0.6870**	0.0282	0.6731	0.0261	0.6532	0.0207	0.6609	0.0253
NR-ER	0.7188	0.0196	**0.7214**	0.0087	0.7108	0.0133	0.7190	0.0107
NR-PPAR-gamma	**0.7614**	0.0212	0.7435	0.0493	0.7538	0.0164	0.7493	0.0171
SR-ARE	0.7297	0.0080	**0.7308**	0.0066	0.7099	0.0118	0.7172	0.0081
SR-ATAD5	**0.7175**	0.0143	0.6896	0.0261	0.6855	0.0295	0.7095	0.0113
SR-HSE	0.7822	0.0097	**0.7833**	0.0116	0.7700	0.0071	0.7745	0.0075
SR-MMP	**0.8120**	0.0075	0.8096	0.0097	0.8099	0.0091	0.8080	0.0091
SR-p53	**0.7518**	0.0198	0.7159	0.0208	0.7417	0.0129	0.7279	0.0113

For comparison, the best results of the SSL-GCN models trained with the entire unlabeled dataset (all) are shown. The complete test performance can be found in the Additional file 1

The bold number denotes the best result among all models (all, close, normal, far) in the corresponding task, the underlined number represents only the best result among models using different similarity levels of unlabeled subsets (close, normal, far)

As shown in Table 6, the optimal model for 7 tasks still belongs to the model trained on the entire unlabeled dataset, SSL-GCN(all). For the remaining 5 tasks, the optimal model for 3 tasks (NR-ER, SR-ARE, SR-HSE) was trained with the close subset, and only for 2 tasks (NR-AR-LBD, NR-Aromatase) the optimal model was trained with the far subset. However, the performance improvement of the SSL-GCN model on these 5 tasks is slight, ranging from 0.0011 to 0.0080, suggesting that the use of close subset and far subset in the SSL process had a limited impact on these models. On the other hand, the use of these similarity-based subsets leads to performance degradation in 7 tasks, with the largest degradation occurring in the NR-AR task, where the average AUC value decreased by 0.0616.

From the perspective of similarity between labeled and unlabeled data, models trained with the close subset tend to perform better than models trained with normal and far subsets. After excluding the performance of SSL-GCN(all) models, 5 SSL-GCN(close) models, 3 SSL-GCN(normal) models, and 3 SSL-GCN(far) models achieved optimal performance on the corresponding task. In addition, the model SSL-GCN(close) outperformed the SSL-GCN(all) model on 3 tasks (NR-ER, SR-ARE, SR-HSE), while this number is 0 for SSL-GCN(normal) model and 2 for SSL-GCN(far) model. Therefore, the performance of SSL-GCN(normal) is the worst among these three types of models; the overall scores of SSL-GCN(near), SSL-GCN(normal), and SSL-GCN(far) on 12 tasks are 0.7417, 0.7388, and 0.7450 respectively, which also indicates this fact.

There are several reasons that lead to this result. First, using unlabeled data in the close subset that is similar to the labeled data allows the semi-supervised learning model to make more accurate predictions about unlabeled data in the early training phase, allowing the model to more accurately generate and update the loss in the early training phase. This enriches the information learned by the model and results in the SSL process generating a better model. Second, using unlabeled data that is dissimilar to the labeled data (far subset) provides additional information for the SSL-GCN model during the semi-supervised learning process. This may improve the generalization ability of the model, which could increase the performance of the model on unseen data. In summary, we believe that using the entire unlabeled dataset and labeled data to train the SSL-GCN model is still the best way to generate the optimal model since the whole unlabeled dataset mixes unlabeled data with different similarities to labeled data.

### Performance comparison of SSL-GCN to the built-in DeepChem methods

The DeepChem package [46] provides some built-in ML methods that can be readily used to generate predictive models for different computational chemistry challenges. Making use of the DeepChem-integrated MoleculeNet datasets [37], we performed experiments to evaluate the performances of the DeepChem models on the Tox21 dataset. The dataset was splitted by scaffold splitting method and all models were initialized with the hyperparameters provided by the DeepChem package. Following the previous experimental procedure, we conducted the training, validation and test processes, and repeated them five times for each model. Here, we benchmark our method by comparing the performance of the SL-GCN and SSL-GCN models in the test set to these DeepChem models in terms of the average ROC-AUC score.

As shown in Table 7, among the 8 DeepChem models, the best one is *kernelsvm*, with an overall score of 0.7, whereas both our models SL-GCN and SSL-GCN beat the best DeepChem model with overall scores of 0.7156 (2% improvement) and 0.7571 (8% improvement), respectively. It should be mentioned that while the *graphconv* model utilizes similar graph convolution technique to our method but its use of different model architecture and molecular feature rendering their model less effective.

**Table 7:** Comparison of our GCN models (SL-GCN and SSL-GCN) and the models constructed using the DeepChem built-in ML methods

Model	Description	Overall score	Std.	Refs.
logreg	Logistic regression model	0.6397	–	[52]
tf	Deep neural network	0.6582	0.0097	[37]
tf-robust	Deep neural network (with bypass layers)	0.6825	0.0056	[53]
rf	Random forest model	0.6618	0.0066	[52]
kernelsvm	Kernel SVM model	0.7000	–	[52]
graphconv	Graph convolutional model	0.6943	0.0043	[54]
irv	Influence relevance voting (IRV) classifier	0.6853	–	[55]
xgb	Xgboost classification model	0.6908	0.0039	[56]
SL-GCN	Supervised GCN model	0.7156	0.0068	This study
SSL-GCN	Semi-supervised GCN model	**0.7571**	0.0084	This study

The overall score is the average ROC-AUC score in predicting the 12 prediction tasks in the test set. The experiments were repeated 5 times

The bold number denotes the best overall score among all models

## Discussion and conclusions

In this work, we attempt to improve compound toxicity prediction using graph convolutional neural network (GCN) and semi-supervised learning (SSL). We choose Mean Teacher [34] as the SSL algorithm to improve the prediction performance of GCN on 12 toxicity prediction tasks from the Tox21 dataset. Meanwhile, we hope to answer two questions about predictive modeling in this research. First, is GCN superior to other more commonly used ML methods? Second, is unlabeled data advantageous for model training?

To this end, we have designed and implemented a GCN model for chemical compounds based on simple physicochemical properties of atoms. Unlike other commonly used chemical fingerprints that represent an entire compound in a one-dimensional feature vector for learning, GCN encodes it into a network of features, where the network resembles bond connectivity in the molecule. Given that structural diversity of a dataset is one of the elements that affect the prediction performance and generalizability of a model, we have used the scaffold splitting approach to divide the dataset into training, validation, and test sets for each prediction task. The Bayesian optimization technique has been used to speed up the process of tuning hyperparameters.

Now, with the GCN model in place, we have trained and optimized the supervised learning SL-GCN models and the semi-supervised learning SSL-GCN models on 12 toxicity prediction tasks. To answer the first question, is GCN superior to other commonly used ML methods? We have trained and optimized toxicity prediction models using 5 conventional ML methods in the supervised learning setting. Our comparative study has revealed that out of the 12 prediction tasks, 5 tasks are better predicted by SL-GCN, 2 tasks are similarly predicted, and 5 tasks are worse by SL-GCN; and the “better” models are not improved by a large margin. Therefore, our experimental result suggests that in the same supervised learning setting, GCN is not superior to conventional ML methods. The answer to this question is a bit disappointing though, as a GCN model is much more complex and expensive to train than the conventional models.

We believe that the bottleneck to improvement is the limitation of available data. Instead of adding more annotated data, which is not always possible or easy, we turn our attention to unlabeled data. Here, we have applied the SSL algorithm, called Mean Teacher (MT), to enhance the performance of the GCN model. Encouragingly, SSL-GCN models consistently outperform their SL-GCN counterparts, with the ROC-AUC scores improving between 1 and 13%. Nonetheless, the amount of unlabeled data required to boost performance has to be determined on a case-by-case basis. We have found that for the prediction of various toxicological endpoints, the appropriate ratios of unlabel-to-label data range from 1 to 4. Larger ratios may improve further, but were not investigated in this study due to limited computational resources. Finally, a comparative analysis of our models with the models from the DeepChem library was done. The findings are that the SL-GCN models are 2 to 12% better than the DeepChem models in terms of ROC-AUC, while the SSL-GCN models are 8 to 18% better. Based on the above results, our answer to the second question, “Is unlabeled data advantageous for model training?”, is therefore yes, and the amount of unlabeled data required to optimize the model is subject to each study.

In many bioinformatics tasks, the size of an annotated dataset is often limited, which complicates the implementation and limits the performance of many ML algorithms. The result of this study suggests that SSL could be applied to other property prediction tasks such as adsorption/distribution/metabolism/excretion (ADME), solubility, binding activity, etc., to improve the predictive ability of model by using unannotated data.

This study does, however, have some limitations that we must point out.

First, the toxicity of a compound is determined by several factors such as chirality and the nature of functional groups. This information requires a more delicate coding approach to avoid information loss during graph conversion. Although there are various well-designed molecular fingerprints or descriptors for conventional ML algorithms that can be used, there is no specific one that is suitable for GCN. Therefore, we have to use the molecule-graph conversion tool from Deep Graph Library (DGL) to convert molecules from SMILES to graphs. However, the graphs converted by this tool only include few basic molecular physicochemical properties. Due to the limited computational power, the running time of the graph convolution layers using the current feature matrix was already very high and adding additional features will certainly cost more time during the model development process. In our future study, it becomes particularly important to increase the diversity of molecular information contained in the feature matrix while limiting the size of the matrix.

Second, the interpretability of our graph convolution model has not been explored. Most researchers consider ML methods with neural networks as a black box. The only factor that can be confirmed during the training or prediction process is the input data, and the prediction results produced by these ML models are unexplainable. Specifically for biomedical ML applications, this limitation has been amplified. Without knowing which part of the compound led to the prediction result, researchers cannot modify the original compounds or select the compounds with better structure to conduct further studies. Therefore, in the next step of our study, we will focus on the interpretability of the graph convolutional neural network.

Third, the activity cliffs problem has not yet been solved in this study. Activity cliffs refer to those chemical compounds that have highly similar structure but different or opposite chemical properties. Although the semi-supervised learning algorithm can use unlabeled data to improve the performance of our GCN model. But nothing comes for free, the basic assumption of the SSL algorithm we implemented is the smoothing assumption, i.e., it assumes that the label function is smooth in high-density areas, so data points located in the same area of the feature space should share the same label. This fundamental assumption makes our model very unreliable in predicting molecules distributed at the edges of high density areas (decision boundary), where most of the molecules with “activity cliffs” are located. Moreover, there is currently no good way for QSAR models to solve the “activity cliff” problem, since the primary assumption of the QSAR model is that similar molecular structure should lead to similar properties [57, 58]. We have already noted that there are some studies [58–62] that attempt to address this problem, and we will follow these studies in our future work.

Finally, our study has exploited the SSL algorithm that is based on the self-ensembling framework. There are other recently proposed SSL algorithms, such as Mixup [63], Interpolation Consistency Training [64], ReMixMatch [65], FixMatch [66], etc. The impact of different SSL algorithms on the toxicity prediction needs further research.

## Supplementary information

**Additional file 1: Table S1.** Full test performances of conventional machine learning models on the 12prediction tasks in 5 repeated experiments. **Table S2.** Full test performances of SL-GCN on the 12 prediction tasks in 5 repeatedexperiments. **Table S3.** Full test performances of SSL-GCN on the 12 prediction tasks in 5 repeatedexperiments. The values in parentheses represent unlabeled data ratios (R_u_). **Table S4.** Full test performance of the SSL-GCN models with different similarity levels of unlabeled subsets (close, normal, far) on the 12 prediction tasks in 5 repeated experiments. **Figure S1.** Details of the ROC curves of the conventional machine learning models in 5repeated experiments on the 12 prediction tasks. **Figure S2.** Details of the ROC curves of the SL-GCN models in 5 repeated experimentson the 12 prediction tasks. **Figure S3.** Details of the ROC curves of the SL-GCN models with different unlabeledratios (R_u_) in 5 repeated experiments on the 12 prediction tasks. **Figure S4.** The distribution of *SS*_*i*_ in 12 similarity domains defined by different cutoff values *C*_*s*_. The *Z* value of these cutoff values range from − 2 to 3.5 with a step size of 0.5.

## Data Availability

All data used in this study comes from MoleculeNet (https://moleculenet.org/). The data and script files for reproducing the experiments can be downloaded from https://github.com/chen709847237/SSL-GCN. The final prediction models we trained in this study are also available.

## References

[1] Llanos EJ, Leal W, Luu DH, Jost J, Stadler PF, Restrepo G (2019). Exploration of the chemical space and its three historical regimes. Proc Natl Acad Sci.

[2] McInnes C (2007). Virtual screening strategies in drug discovery. Curr Opin Chem Biol.

[3] Kubinyi H, Mannhold R, Timmerman H (2008). Virtual screening for bioactive molecules.

[4] Dean A, Lewis S (2006). Screening: methods for experimentation in industry, drug discovery, and genetics.

[5] Oprea TI, Matter H (2004). Integrating virtual screening in lead discovery. Curr Opin Chem Biol.

[6] Bailey J, Balls M (2019). Recent efforts to elucidate the scientific validity of animal-based drug tests by the pharmaceutical industry, pro-testing lobby groups, and animal welfare organisations. BMC Med Ethics.

[7] Pu L, Naderi M, Liu T, Wu H-C, Mukhopadhyay S, Brylinski M (2019). e toxpred: a machine learning-based approach to estimate the toxicity of drug candidates. BMC Pharmacol Toxicol.

[8] Raies AB, Bajic VB (2016). In silico toxicology: computational methods for the prediction of chemical toxicity. Wiley Interdiscipl Rev Comput Mol Sci.

[9] McKinney JD, Richard A, Waller C, Newman MC, Gerberick F (2000). The practice of structure activity relationships (SAR) in toxicology. Toxicol Sci.

[10] Roy K, Kar S, Das R, Roy K, Kar S, Das RN (2015). Chapter 7—validation of qsar models. Understanding the basics of QSAR for applications in pharmaceutical sciences and risk assessment.

[11] Wu Y, Wang G (2018). Machine learning based toxicity prediction: from chemical structural description to transcriptome analysis. Int J Mol Sci.

[12] Idakwo G, Luttrell J, Chen M, Hong H, Zhou Z, Gong P, Zhang C (2018). A review on machine learning methods for in silico toxicity prediction. J Environ Sci Health Part C.

[13] Yang H, Sun L, Li W, Liu G, Tang Y (2018). In silico prediction of chemical toxicity for drug design using machine learning methods and structural alerts. Front Chem.

[14] Kearnes S, McCloskey K, Berndl M, Pande V, Riley P (2016). Molecular graph convolutions: moving beyond fingerprints. J Comput Aided Mol Design.

[15] Li J, Cai D, He X (2017) Learning graph-level representation for drug discovery. arXiv preprint arXiv:1709.03741

[16] Wang F, Yang JF, Wang MY, Jia CY, Shi XX, Hao GF, Yang GF (2020). Graph attention convolutional neural network model for chemical poisoning of honey bees’ prediction. Sci Bull.

[17] Lusci A, Pollastri G, Baldi P (2013). Deep architectures and deep learning in chemoinformatics: the prediction of aqueous solubility for drug-like molecules. J Chem Inform Model.

[18] Feinberg EN, Sur D, Wu Z, Husic BE, Mai H, Li Y, Sun S, Yang J, Ramsundar B, Pande VS (2018). Potentialnet for molecular property prediction. ACS Central Sci.

[19] Portugal I, Alencar P, Cowan D (2018). The use of machine learning algorithms in recommender systems: a systematic review. Expert Syst Appl.

[20] Altae-Tran H, Ramsundar B, Pappu AS, Pande V (2017). Low data drug discovery with one-shot learning. ACS Central Sci.

[21] Rao B, Zhang L, Zhang G (2020). Acp-gcn: the identification of anticancer peptides based on graph convolution networks. IEEE Access.

[22] Li G, Muller M, Thabet A, Ghanem B (2019) Deepgcns: can gcns go as deep as cnns? In: Proceedings of the IEEE/CVF international conference on computer vision, pp 9267–9276

[23] Tang L, Liu H (2009) Relational learning via latent social dimensions. In: Proceedings of the 15th ACM SIGKDD international conference on knowledge discovery and data mining, pp 817–826

[24] Marcheggiani D, Titov I (2017) Encoding sentences with graph convolutional networks for semantic role labeling. arXiv preprint arXiv:1703.04826

[25] Bastings J, Titov I, Aziz W, Marcheggiani D, Sima’an K (2017) Graph convolutional encoders for syntax-aware neural machine translation. arXiv preprint arXiv:1704.04675

[26] Ying R, He R, Chen K, Eksombatchai P, Hamilton WL, Leskovec J (2018) Graph convolutional neural networks for web-scale recommender systems. In: Proceedings of the 24th ACM SIGKDD international conference on knowledge discovery & data mining, pp 974–983

[27] Monti F, Bronstein MM, Bresson X (2017) Geometric matrix completion with recurrent multi-graph neural networks. arXiv preprint arXiv:1704.06803

[28] Kipf TN, Welling M (2016) Semi-supervised classification with graph convolutional networks. arXiv preprint arXiv:1609.02907

[29] Chen J, Siu SW (2020). Machine learning approaches for quality assessment of protein structures. Biomolecules.

[30] Kotsiantis SB, Zaharakis I, Pintelas P (2007). Supervised machine learning: a review of classification techniques. Emerg Artif Intell Appl Comput Eng.

[31] Cui W, Liu Y, Li Y, Guo M, Li Y, Li X, Wang T, Zeng X, Ye, C (2019) Semi-supervised brain lesion segmentation with an adapted mean teacher model. In: International conference on information processing in medical imaging. Springer, pp 554–565

[32] Van Engelen JE, Hoos HH (2020). A survey on semi-supervised learning. Mach Learn.

[33] Rasmus A, Valpola H, Honkala M, Berglund M, Raiko T (2015) Semi-supervised learning with ladder networks. arXiv preprint arXiv:1507.02672

[34] Tarvainen A, Valpola H (2017) Mean teachers are better role models: Weight-averaged consistency targets improve semi-supervised deep learning results. arXiv preprint arXiv:1703.01780

[35] Laine S, Aila T (2016) Temporal ensembling for semi-supervised learning. arXiv preprint arXiv:1610.02242

[36] Kingma DP, Ba J (2014) Adam: a method for stochastic optimization. arXiv preprint arXiv:1412.6980

[37] Wu Z, Ramsundar B, Feinberg EN, Gomes J, Geniesse C, Pappu AS, Leswing K, Pande V (2018). Moleculenet: a benchmark for molecular machine learning. Chem Sci.

[38] Sheridan RP (2013). Time-split cross-validation as a method for estimating the goodness of prospective prediction. J Chem Inform Model.

[39] Bemis GW, Murcko MA (1996). The properties of known drugs. 1. molecular frameworks. J Med Chem.

[40] RDKit: Open-Source Cheminformatics Software (2006). https://www.rdkit.org/ Accessed 14 July 2021

[41] Wang M, Yu L, Zheng D, Gan Q, Gai Y, Ye Z, Li M, Zhou J, Huang Q, Ma C et al. (2019) Deep graph library: towards efficient and scalable deep learning on graphs

[42] Bergstra J, Yamins D, Cox D (2013) Making a science of model search: Hyperparameter optimization in hundreds of dimensions for vision architectures. In: International conference on machine learning, pp 115–123. PMLR

[43] DGL: Deep Graph Library (2018). https://github.com/dmlc/dgl. Accessed 14 July 2021

[44] DGL-LifeSci (2020). https://github.com/awslabs/dgl-lifesci. Accessed 14 July 2021

[45] Hyperopt: Distributed Hyperparameter Optimization (2018). https://github.com/hyperopt/hyperopt. Accessed 14 July 2021

[46] Ramsundar B, Eastman P, Walters P, Pande V, Leswing K, Wu Z (2019) Deep learning for the life sciences. O’Reilly Media, 1005 Gravenstein Highway North, Sebastopol, CA 95472, USA

[47] DeepChem (2015). https://github.com/deepchem/deepchem. Accessed 14 July 2021

[48] Mean teachers are better role models (2018). https://github.com/CuriousAI/mean-teacher. Accessed 17 Oct 2021

[49] Rogers D, Hahn M (2010). Extended-connectivity fingerprints. J Chem Inform Model.

[50] Tropsha A, Gramatica P, Gombar VK (2003). The importance of being earnest: validation is the absolute essential for successful application and interpretation of qspr models. QSAR Combinatorial Sci.

[51] Shen M, LeTiran A, Xiao Y, Golbraikh A, Kohn H, Tropsha A (2002). Quantitative structure-activity relationship analysis of functionalized amino acid anticonvulsant agents using k nearest neighbor and simulated annealing pls methods. J Med Chem.

[52] Pedregosa F, Varoquaux G, Gramfort A, Michel V, Thirion B, Grisel O, Blondel M, Prettenhofer P, Weiss R, Dubourg V, Vanderplas J, Passos A, Cournapeau D, Brucher M, Perrot M, Duchesnay E (2011). Scikit-learn: machine learning in Python. J Mach Learning Res.

[53] Ramsundar B, Liu B, Wu Z, Verras A, Tudor M, Sheridan RP, Pande V (2017). Is multitask deep learning practical for pharma?. J Chem Inform Model.

[54] Duvenaud D, Maclaurin D, Aguilera-Iparraguirre J, Gómez-Bombarelli R, Hirzel T, Aspuru-Guzik A, Adams RP (2015) Convolutional networks on graphs for learning molecular fingerprints. arXiv preprint arXiv:1509.09292

[55] Swamidass SJ, Azencott C-A, Lin T-W, Gramajo H, Tsai S-C, Baldi P (2009). Influence relevance voting: an accurate and interpretable virtual high throughput screening method. J Chem Inform Model.

[56] Chen T, Guestrin C (2016) Xgboost: A scalable tree boosting system. In: Proceedings of the 22nd Acm Sigkdd international conference on knowledge discovery and data mining, pp 785–794

[57] Maggiora GM (2006). On outliers and activity cliffs why QSAR often disappoints.

[58] Kim H, Kim E, Lee I, Bae B, Park M, Nam H (2020). Artificial intelligence in drug discovery: a comprehensive review of data-driven and machine learning approaches. Biotechnol Bioprocess Eng.

[59] Kohonen P, Parkkinen JA, Willighagen EL, Ceder R, Wennerberg K, Kaski S, Grafström RC (2017). A transcriptomics data-driven gene space accurately predicts liver cytopathology and drug-induced liver injury. Nat Commun.

[60] Rueda-Zárate HA, Imaz-Rosshandler I, Cárdenas-Ovando RA, Castillo-Fernández JE, Noguez-Monroy J, Rangel-Escareño C (2017). A computational toxicogenomics approach identifies a list of highly hepatotoxic compounds from a large microarray database. PLoS ONE.

[61] Su R, Wu H, Xu B, Liu X, Wei L (2018). Developing a multi-dose computational model for drug-induced hepatotoxicity prediction based on toxicogenomics data. IEEE/ACM Trans Comput Biol Bioinformatics.

[62] Blaschke T, Feldmann C, Bajorath J (2021). Prediction of promiscuity cliffs using machine learning. Mol Inform.

[63] Zhang H, Cisse M, Dauphin YN, Lopez-Paz D (2017) mixup: beyond empirical risk minimization. arXiv preprint arXiv:1710.09412

[64] Verma V, Kawaguchi K, Lamb A, Kannala J, Bengio Y, Lopez-Paz D (2019) Interpolation consistency training for semi-supervised learning. arXiv preprint arXiv:1903.03825 10.1016/j.neunet.2021.10.008 34735894

[65] Berthelot D, Carlini N, Cubuk ED, Kurakin A, Sohn K, Zhang H, Raffel C (2019) Remixmatch: semi-supervised learning with distribution alignment and augmentation anchoring. arXiv preprint arXiv:1911.09785

[66] Sohn K, Berthelot D, Li C-L, Zhang Z, Carlini N, Cubuk ED, Kurakin A, Zhang H, Raffel C (2020) Fixmatch: simplifying semi-supervised learning with consistency and confidence. arXiv preprint arXiv:2001.07685

