# Supplementary material for: Chemical toxicity prediction based on semi-supervised learning and graph convolutional neural network
Source: J Cheminform. 2021 Nov 27;13:93. doi: 10.1186/s13321-021-00570-8 (PMC8627024; doi:10.1186/s13321-021-00570-8)

SUPPLEMENTARY MATERIAL

Chemical Toxicity Prediction Based on Semi-supervised Learning and Graph Convolutional Neural Network

Jiarui Chen[1], Yain-Whar Si[1], Chon-Wai Un[1], and Shirley W. I. Siu*[123]

[1]Department of Computer and Information Science, University of Macau,

Avenida da Universidade, Taipa, 999078 Macau, China

[2]Institute of Science and Environment, University of Saint Joseph,

Rua de Londres 106, 999078 Macau, China

[3]School of Pharmaceutical Sciences, Universiti Sains Malaysia,

11800 USM Penang, Malaysia

Corresponding Author

E-mail: shirleysiu@um.edu.mo **Phone:** +853 8822-4452 **Fax:** +853 8822-2426

Table S1. Full test performances of conventional machine learning models on the 12 prediction tasks in 5 repeated experiments.

Task	Model	Test AUC	Average AUC	Std.
NR-AR-LBD	KNN	0.6955	0.6955	-
		0.6955		
		0.6955		
		0.6955		
		0.6955		
	NN	0.6704	0.6671	0.0244
		0.6982		
		0.6252		
		0.6812		
		0.6601		
	RF	0.7190	0.7323	0.0267
		0.7275		
		0.7141		
		0.7145		
		0.7841		
	SVM	0.6795	0.6795	-
		0.6795		
		0.6795		
		0.6795		
		0.6795		
	XGBoost	0.6784	0.6784	-
		0.6784		
		0.6784		
		0.6784		
		0.6784		
NR-AR	KNN	0.6527	0.6527	-
		0.6527		
		0.6527		
		0.6527		
		0.6527		
	NN	0.6742	0.6806	0.0088
		0.6864		
		0.6943		
		0.6782		
		0.6699		
	RF	0.6399	0.6836	0.0266
		0.6969		

		0.7018		
		0.6663		
		0.7121		
	SVM	0.7193	0.7193	-
		0.7193		
		0.7193		
		0.7193		
		0.7193		
	XGBoost	0.6818	0.6818	-
		0.6818		
		0.6818		
		0.6818		
		0.6818		
NR-AhR	KNN	0.7639	0.7639	-
		0.7639		
		0.7639		
		0.7639		
		0.7639		
	NN	0.7723	0.7628	0.0177
		0.7699		
		0.7648		
		0.7288		
		0.7792		
	RF	0.8256	0.8243	0.0074
		0.8312		
		0.8193		
		0.8327		
		0.8130		
	SVM	0.7794	0.7794	-
		0.7794		
		0.7794		
		0.7794		
		0.7794		
	XGBoost	0.8287	0.8287	-
		0.8287		
		0.8287		
		0.8287		
		0.8287		
NR-Aromatase	KNN	0.5576	0.5576	-
		0.5576		
		0.5576		

		0.5576		
		0.5576		
	NN	0.5112	0.5127	0.0772
		0.5643		
		0.3806		
		0.4981		
		0.6102		
	RF	0.6978	0.6900	0.0092
		0.6973		
		0.6855		
		0.6739		
		0.6953		
	SVM	0.6873	0.6873	-
		0.6873		
		0.6873		
		0.6873		
		0.6873		
	XGBoost	0.7106	0.7106	-
		0.7106		
		0.7106		
		0.7106		
		0.7106		
NR-ER-LBD	KNN	0.6191	0.6191	-
		0.6191		
		0.6191		
		0.6191		
		0.6191		
	NN	0.7072	0.5387	0.1171
		0.5379		
		0.4452		
		0.6217		
		0.3810		
	RF	0.6105	0.6169	0.0300
		0.5815		
		0.5908		
		0.6390		
		0.6627		
	SVM	0.6078	0.6078	-
		0.6078		
		0.6078		
		0.6078		

		0.6078		
	XGBoost	0.6250	0.6250	-
		0.6250		
		0.6250		
		0.6250		
		0.6250		
NR-ER	KNN	0.6597	0.6597	-
		0.6597		
		0.6597		
		0.6597		
		0.6597		
	NN	0.6431	0.6549	0.0162
		0.6350		
		0.6505		
		0.6798		
		0.6659		
	RF	0.6428	0.6316	0.0080
		0.6341		
		0.6365		
		0.6209		
		0.6245		
	SVM	0.6126	0.6126	-
		0.6126		
		0.6126		
		0.6126		
		0.6126		
	XGBoost	0.6745	0.6745	-
		0.6745		
		0.6745		
		0.6745		
		0.6745		
NR-PPAR-gamma	KNN	0.6182	0.6182	-
		0.6182		
		0.6182		
		0.6182		
		0.6182		
	NN	0.6023	0.5558	0.0736
		0.6403		
		0.4907		
		0.4475		
		0.5975		

	RF	0.7276	0.7135	0.0258
		0.7454		
		0.6844		
		0.7251		
		0.6812		
	SVM	0.6454	0.6454	-
		0.6454		
		0.6454		
		0.6454		
		0.6454		
	XGBoost	0.6414	0.6414	-
		0.6414		
		0.6414		
		0.6414		
		0.6414		
SR-ARE	KNN	0.6366	0.6366	-
		0.6366		
		0.6366		
		0.6366		
		0.6366		
	NN	0.5315	0.5656	0.0251
		0.5822		
		0.5870		
		0.5388		
		0.5887		
	RF	0.6593	0.6603	0.0018
		0.6593		
		0.6631		
		0.6616		
		0.6581		
	SVM	0.6843	0.6843	-
		0.6843		
		0.6843		
		0.6842		
		0.6843		
	XGBoost	0.6640	0.6640	-
		0.6640		
		0.6640		
		0.6640		
		0.6640		
SR-ATAD5	KNN	0.5866	0.5866	-

		0.5866		
		0.5866		
		0.5866		
		0.5866		
	NN	0.6359	0.6240	0.0537
		0.6604		
		0.6302		
		0.5211		
		0.6726		
	RF	0.7160	0.6928	0.0189
		0.7095		
		0.6747		
		0.6675		
		0.6957		
	SVM	0.6546	0.6546	-
		0.6546		
		0.6546		
		0.6546		
		0.6546		
	XGBoost	0.6841	0.6841	-
		0.6841		
		0.6841		
		0.6841		
		0.6841		
SR-HSE	KNN	0.6574	0.6574	-
		0.6574		
		0.6574		
		0.6574		
		0.6574		
	NN	0.6421	0.6143	0.0222
		0.6010		
		0.6346		
		0.5811		
		0.6126		
	RF	0.6926	0.6852	0.0131
		0.6847		
		0.6847		
		0.6623		
		0.7018		
	SVM	0.6858	0.6858	-
		0.6858		

		0.6858		
		0.6858		
		0.6858		
	XGBoost	0.6647	0.6647	-
		0.6647		
		0.6647		
		0.6647		
		0.6647		
SR-MMP	KNN	0.7057	0.7057	-
		0.7057		
		0.7057		
		0.7057		
		0.7057		
	NN	0.7041	0.6551	0.0612
		0.6987		
		0.5366		
		0.6729		
		0.6630		
	RF	0.7706	0.7818	0.0065
		0.7809		
		0.7864		
		0.7899		
		0.7811		
	SVM	0.7794	0.7794	-
		0.7794		
		0.7794		
		0.7794		
		0.7794		
	XGBoost	0.7656	0.7656	-
		0.7656		
		0.7656		
		0.7656		
		0.7656		
SR-p53	KNN	0.6778	0.6778	-
		0.6778		
		0.6778		
		0.6778		
		0.6778		
	NN	0.5892	0.5963	0.0075
		0.5992		
		0.5914		

		0.5920		
		0.6101		
	RF	0.7114	0.7263	0.0130
		0.7173		
		0.7199		
		0.7412		
		0.7440		
	SVM	0.7081	0.7051	-
		0.7007		
		0.7071		
		0.7081		
		0.7016		
	XGBoost	0.6942	0.6942	-
		0.6942		
		0.6942		
		0.6942		
		0.6942		

Table S2. Full test performances of SL-GCN on the 12 prediction tasks in 5 repeated experiments.

Task	Model	Test AUC	Average AUC	Std.
NR-AR-LBD	SL-GCN	0.6297	0.6783	0.0269
		0.7012		
		0.7046		
		0.6829		
		0.6729		
NR-AR	SL-GCN	0.7104	0.7157	0.0367
		0.7607		
		0.7291		
		0.7286		
		0.6500		
NR-AhR	SL-GCN	0.8150	0.8260	0.0055
		0.8292		
		0.8291		
		0.8298		
		0.8283		
NR-Aromatase	SL-GCN	0.7123	0.7092	0.0167
		0.7034		
		0.7308		
		0.7182		
		0.6809		
NR-ER-LBD	SL-GCN	0.6076	0.6340	0.0161
		0.6320		
		0.6542		
		0.6464		
		0.6290		
NR-ER	SL-GCN	0.6927	0.6899	0.0160
		0.7102		
		0.6741		
		0.6691		
		0.7029		
NR-PPAR-gamma	SL-GCN	0.6804	0.6753	0.0278
		0.6259		
		0.7124		
		0.6773		
		0.6802		
SR-ARE	SL-GCN	0.7116	0.7134	0.0137
		0.7099		

		0.7158		
		0.7360		
		0.6934		
SR-ATAD5	SL-GCN	0.6820	0.6850	0.0223
		0.6645		
		0.7119		
		0.6576		
		0.7090		
SR-HSE	SL-GCN	0.7595	0.7644	0.0096
		0.7634		
		0.7564		
		0.7830		
		0.7595		
SR-MMP	SL-GCN	0.7868	0.7988	0.0066
		0.8023		
		0.7993		
		0.8068		
		0.7989		
SR-p53	SL-GCN	0.6923	0.6970	0.0253
		0.7254		
		0.6709		
		0.6701		
		0.7273		

Table S3. Full test performances of SSL-GCN on the 12 prediction tasks in 5 repeated experiments. The values in parentheses represent unlabeled data ratios (R_u).

Task	Model	Test AUC	Average AUC	Std.
NR-AR-LBD	SSL-GCN (0.5)	0.7477	0.7417	0.0105
		0.7479		
		0.7253		
		0.7337		
		0.7533		
	SSL-GCN (1.0)	0.6997	0.7333	0.0401
		0.7775		
		0.7634		
		0.7531		
		0.6727		
	**SSL-GCN (2.0)**	0.7919	**0.7647**	**0.0279**
		0.7821		
		0.7865		
		0.7401		
		0.7226		
	SSL-GCN (3.0)	0.7176	0.7377	0.0145
		0.7391		
		0.7314		
		0.7622		
		0.7380		
	SSL-GCN (4.0)	0.7572	0.7477	0.0135
		0.7272		
		0.7514		
		0.7646		
		0.7375		
NR-AR	SSL-GCN (0.5)	0.6974	0.7550	0.0483
		0.7920		
		0.7127		
		0.8264		
		0.7464		
	SSL-GCN (1.0)	0.7320	0.7858	0.0357
		0.8163		
		0.8251		
		0.7567		
		0.7985		
	SSL-GCN (2.0)	0.6988	0.7512	0.0358
		0.7665		

		0.7420		
		0.7409		
		0.8079		
	SSL-GCN (3.0)	0.6736	0.7412	0.0659
		0.7379		
		0.8351		
		0.6661		
		0.7931		
	**SSL-GCN (4.0)**	0.8217	**0.7967**	**0.0251**
		0.7604		
		0.8033		
		0.7748		
		0.8230		
NR-AhR	SSL-GCN (0.5)	0.8252	0.8161	0.0121
		0.8056		
		0.8351		
		0.8035		
		0.8119		
	SSL-GCN (1.0)	0.8109	0.8295	0.0129
		0.8185		
		0.8341		
		0.8436		
		0.8412		
	SSL-GCN (2.0)	0.8317	0.8287	0.0072
		0.8313		
		0.8286		
		0.8152		
		0.8366		
	**SSL-GCN (3.0)**	0.8206	**0.8303**	**0.0055**
		0.8357		
		0.8281		
		0.8341		
		0.8335		
	SSL-GCN (4.0)	0.8103	0.8224	0.0090
		0.8249		
		0.8190		
		0.8220		
		0.8376		
NR-Aromatase	SSL-GCN (0.5)	0.7144	0.7202	0.0057
		0.7225		
		0.7290		

		0.7214		
		0.7137		
	SSL-GCN (1.0)	0.7284	0.7306	0.0156
		0.7210		
		0.7245		
		0.7610		
		0.7180		
	SSL-GCN (2.0)	0.7296	0.7232	0.0040
		0.7182		
		0.7202		
		0.7226		
		0.7257		
	SSL-GCN (3.0)	0.7431	0.7287	0.0082
		0.7309		
		0.7225		
		0.7274		
		0.7196		
	**SSL-GCN (4.0)**	0.7341	**0.7337**	**0.0057**
		0.7376		
		0.7417		
		0.7295		
		0.7257		
NR-ER-LBD	SSL-GCN (0.5)	0.6121	0.6623	0.0330
		0.6563		
		0.7032		
		0.6467		
		0.6932		
	SSL-GCN (1.0)	0.7057	0.6794	0.0411
		0.6234		
		0.6656		
		0.6594		
		0.7428		
	SSL-GCN (2.0)	0.6785	0.6772	0.0161
		0.7038		
		0.6682		
		0.6549		
		0.6804		
	SSL-GCN (3.0)	0.6755	0.6662	0.0250
		0.6207		
		0.6831		
		0.6913		

		0.6599		
	**SSL-GCN (4.0)**	0.7387	**0.6870**	**0.0282**
		0.6602		
		0.6934		
		0.6756		
		0.6664		
NR-ER	**SSL-GCN (0.5)**	0.7123	**0.7188**	**0.0196**
		0.7219		
		0.7465		
		0.7247		
		0.6865		
	SSL-GCN (1.0)	0.7297	0.7114	0.0179
		0.7057		
		0.7300		
		0.6818		
		0.7085		
	SSL-GCN (2.0)	0.7016	0.7039	0.0124
		0.7188		
		0.7155		
		0.6847		
		0.6980		
	SSL-GCN (3.0)	0.7212	0.7113	0.0083
		0.6998		
		0.7115		
		0.7041		
		0.7192		
	SSL-GCN (4.0)	0.7234	0.7166	0.0137
		0.7076		
		0.7201		
		0.6955		
		0.7354		
NR-PPAR-gamma	SSL-GCN (0.5)	0.7248	0.7267	0.0210
		0.7165		
		0.7398		
		0.7567		
		0.6949		
	**SSL-GCN (1.0)**	0.7753	**0.7614**	**0.0212**
		0.7201		
		0.7776		
		0.7638		
		0.7699		

	SSL-GCN (2.0)	0.7356	0.7491	0.0201
		0.7565		
		0.7416		
		0.7267		
		0.7843		
	SSL-GCN (3.0)	0.7554	0.7429	0.0177
		0.7446		
		0.7156		
		0.7321		
		0.7660		
	SSL-GCN (4.0)	0.7310	0.7456	0.0223
		0.7298		
		0.7675		
		0.7218		
		0.7769		
SR-ARE	SSL-GCN (0.5)	0.7292	0.7241	0.0065
		0.7296		
		0.7252		
		0.7118		
		0.7249		
	SSL-GCN (1.0)	0.7232	0.7288	0.0063
		0.7338		
		0.7281		
		0.7212		
		0.7378		
	**SSL-GCN (2.0)**	0.7382	**0.7297**	**0.0080**
		0.7252		
		0.7401		
		0.7250		
		0.7198		
	SSL-GCN (3.0)	0.7382	0.7277	0.0067
		0.7300		
		0.7178		
		0.7282		
		0.7245		
	SSL-GCN (4.0)	0.7182	0.7243	0.0114
		0.7327		
		0.7104		
		0.7420		
		0.7180		
SR-ATAD5		0.7182	**0.7119**	**0.0080**

	**SSL-GCN (0.5)**	0.7159		
		0.7060		
		0.6990		
		0.7195		
	SSL-GCN (1.0)	0.6925	0.7061	0.0245
		0.7329		
		0.6948		
		0.7363		
		0.6737		
	SSL-GCN (2.0)	0.6846	0.7096	0.0139
		0.7241		
		0.7055		
		0.7191		
		0.7142		
	SSL-GCN (3.0)	0.7302	0.7175	0.0143
		0.6959		
		0.7341		
		0.7195		
		0.7071		
	SSL-GCN (4.0)	0.7091	0.7077	0.0162
		0.6878		
		0.7255		
		0.7250		
		0.6905		
SR-HSE	SSL-GCN (0.5)	0.7916	0.7636	0.0239
		0.7202		
		0.7604		
		0.7759		
		0.7699		
	SSL-GCN (1.0)	0.7680	0.7678	0.0080
		0.7562		
		0.7812		
		0.7673		
		0.7662		
	**SSL-GCN (2.0)**	0.7769	**0.7822**	**0.0097**
		0.7819		
		0.7961		
		0.7885		
		0.7676		
	SSL-GCN (3.0)	0.7771	0.7731	0.0098
		0.7654		

		0.7764		
		0.7593		
		0.7875		
	SSL-GCN (4.0)	0.7760	0.7700	0.0066
		0.7575		
		0.7709		
		0.7703		
		0.7751		
SR-MMP	**SSL-GCN (0.5)**	0.8097	**0.8120**	**0.0075**
		0.8107		
		0.8001		
		0.8167		
		0.8225		
	SSL-GCN (1.0)	0.7982	0.8035	0.0061
		0.8060		
		0.8025		
		0.8138		
		0.7970		
	SSL-GCN (2.0)	0.8079	0.8100	0.0033
		0.8052		
		0.8142		
		0.8097		
		0.8131		
	SSL-GCN (3.0)	0.8121	0.8031	0.0088
		0.8051		
		0.8111		
		0.7990		
		0.7881		
	SSL-GCN (4.0)	0.7990	0.8081	0.0078
		0.8044		
		0.8059		
		0.8224		
		0.8088		
SR-p53	SSL-GCN (0.5)	0.7265	0.7291	0.0114
		0.7484		
		0.7331		
		0.7255		
		0.7144		
	SSL-GCN (1.0)	0.7384	0.7401	0.0203
		0.7037		
		0.7599		

		0.7545		
		0.7474		
	**<u>SSL-GCN (2.0)</u>**	0.7485	**<u>0.7518</u>**	**<u>0.0198</u>**
		0.7744		
		0.7449		
		0.7211		
		0.7712		
	SSL-GCN (3.0)	0.7383	0.7359	0.0147
		0.7524		
		0.7261		
		0.7524		
		0.7149		
	SSL-GCN (4.0)	0.7271	0.7434	0.0126
		0.7563		
		0.7579		
		0.7311		
		0.7463		

Table S4. Full test performance of the SSL-GCN models with different similarity levels of unlabeled subsets (close, normal, far) on the 12 prediction tasks in 5 repeated experiments.

NR-AR-LBD	close	0.7323	0.7353	0.0353
		0.7831		
		0.7567		
		0.7041		
		0.7000		
	normal	0.7351	0.7410	0.0210
		0.7450		
		0.7581		
		0.7079		
		0.7591		
	far	0.7736	0.7726	0.0242
		0.7890		
		0.7371		
		0.7636		
		0.7998		
NR-AR	close	0.6509	0.7398	0.0594
		0.7728		
		0.7108		
		0.8001		
		0.7645		
	normal	0.7205	0.7389	0.0401
		0.6792		
		0.7478		
		0.7686		
		0.7786		
	far	0.7134	0.7351	0.0357
		0.7405		
		0.7834		
		0.6895		
		0.7489		
NR-AhR	close	0.8248	0.8261	0.0076
		0.8335		
		0.8137		
		0.8287		
		0.8299		
	normal	0.8397	0.8292	0.0080
		0.8306		

		0.8327		
		0.8187		
		0.8244		
	far	0.8209	0.8278	0.0055
		0.8310		
		0.8321		
		0.8230		
		0.8323		
NR-Aromatase	close	0.7341	0.7318	0.0082
		0.7301		
		0.7302		
		0.7436		
		0.7209		
	normal	0.7275	0.7222	0.0131
		0.7367		
		0.7215		
		0.7012		
		0.7243		
	far	0.7352	0.7382	0.0145
		0.7312		
		0.7299		
		0.7309		
		0.7639		
NR-ER-LBD	close	0.6941	0.6731	0.0261
		0.6420		
		0.7050		
		0.6675		
		0.6571		
	normal	0.6716	0.6532	0.0207
		0.6365		
		0.6549		
		0.6748		
		0.6282		
	far	0.6362	0.6609	0.0253
		0.6786		
		0.6313		
		0.6724		
		0.6857		
NR-ER	close	0.7194	0.7214	0.0087
		0.7109		
		0.7208		

		0.7208		
		0.7352		
	normal	0.7105	0.7108	0.0133
		0.7331		
		0.7086		
		0.7002		
		0.7013		
	far	0.7171	0.7190	0.0107
		0.7268		
		0.7327		
		0.7124		
		0.7062		
NR-PPAR-gamma	close	0.7049	0.7435	0.0493
		0.6975		
		0.7230		
		0.8054		
		0.7868		
	normal	0.7506	0.7538	0.0164
		0.7793		
		0.7370		
		0.7586		
		0.7433		
	far	0.7615	0.7493	0.0171
		0.7628		
		0.7291		
		0.7608		
		0.7320		
SR-ARE	close	0.7386	0.7308	0.0066
		0.7255		
		0.7346		
		0.7327		
		0.7225		
	normal	0.7297	0.7099	0.0118
		0.7023		
		0.7002		
		0.7070		
		0.7105		
	far	0.7152	0.7172	0.0081
		0.7064		
		0.7247		
		0.7258		

		0.7140		
SR-ATAD5	close	0.7250	0.6896	0.0261
		0.6592		
		0.6863		
		0.7049		
		0.6724		
	normal	0.7125	0.6855	0.0295
		0.7173		
		0.6644		
		0.6839		
		0.6495		
	far	0.7156	0.7095	0.0113
		0.7108		
		0.7083		
		0.6914		
		0.7214		
SR-HSE	close	0.7641	0.7833	0.0116
		0.7890		
		0.7832		
		0.7951		
		0.7850		
	normal	0.7619	0.7700	0.0071
		0.7794		
		0.7646		
		0.7694		
		0.7745		
	far	0.7876	0.7745	0.0075
		0.7694		
		0.7711		
		0.7735		
		0.7707		
SR-MMP	close	0.8163	0.8096	0.0097
		0.7936		
		0.8161		
		0.8147		
		0.8070		
	normal	0.7987	0.8099	0.0091
		0.8131		
		0.8065		
		0.8233		
		0.8077		

	far	0.8019	0.8080	0.0091
		0.8131		
		0.7964		
		0.8093		
		0.8196		
SR-p53	close	0.7118	0.7159	0.0208
		0.7463		
		0.7220		
		0.7099		
		0.6893		
	normal	0.7390	0.7417	0.0129
		0.7232		
		0.7457		
		0.7591		
		0.7415		
	far	0.7436	0.7279	0.0113
		0.7182		
		0.7344		
		0.7166		
		0.7269		

Figure S1. Details of the ROC curves of the conventional machine learning models in 5 repeated experiments on the 12 prediction tasks.

CM Models - NR-AhR

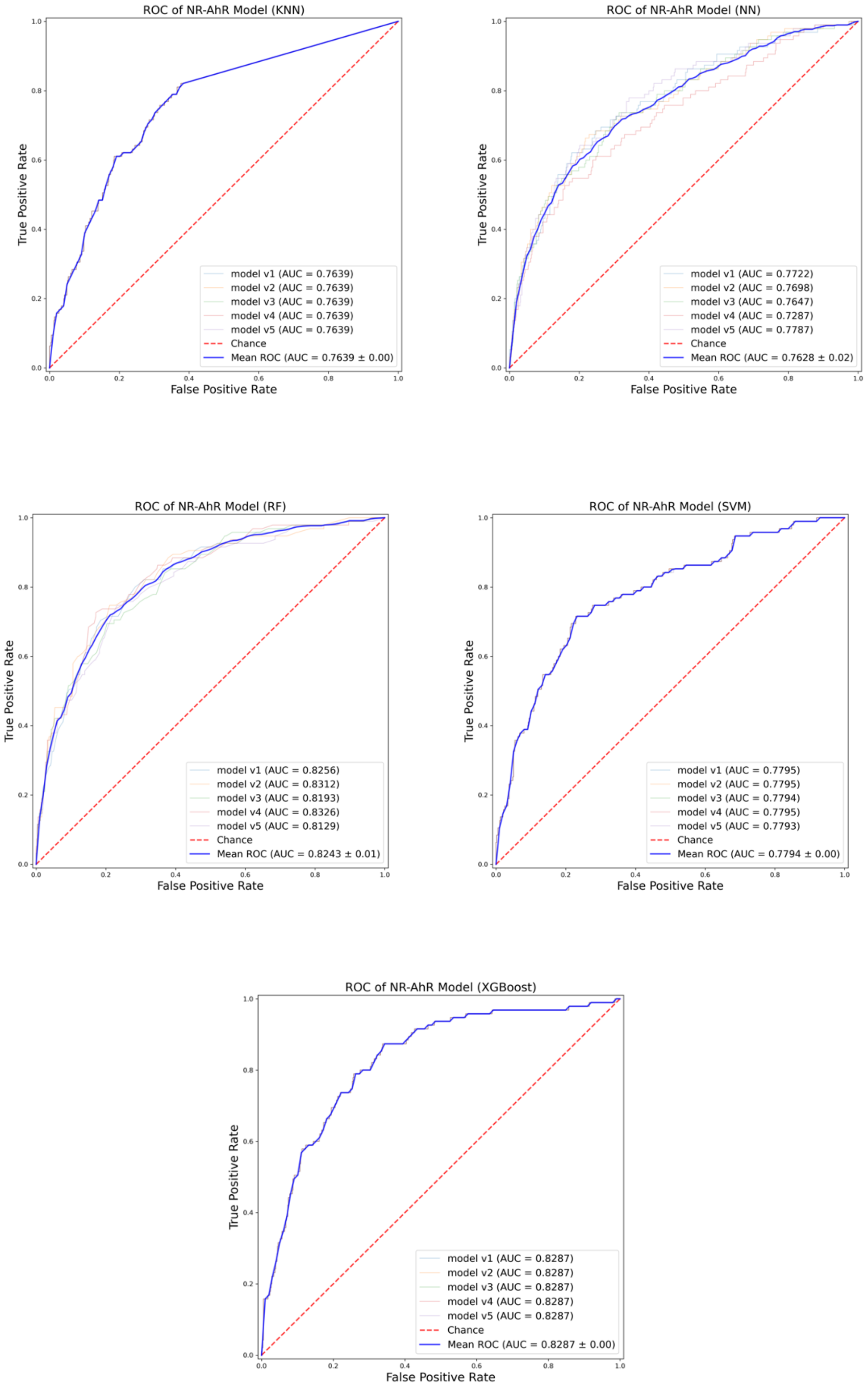

CM Models - NR-AR-LBD

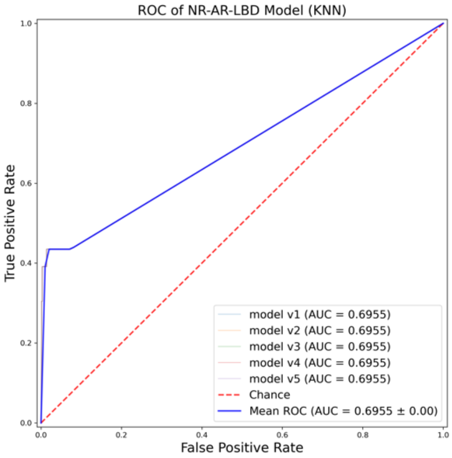

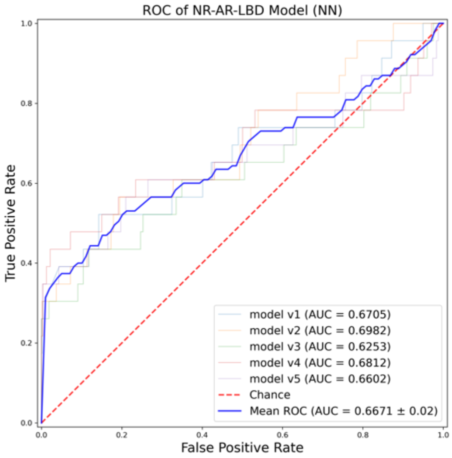

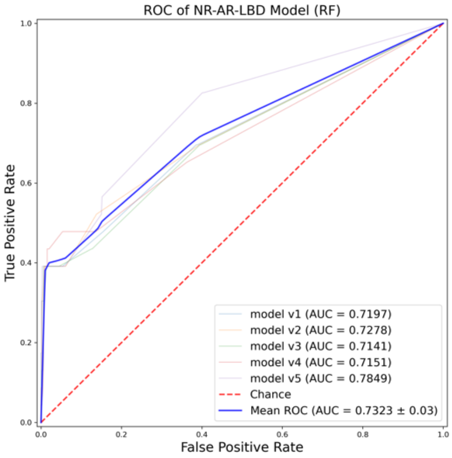

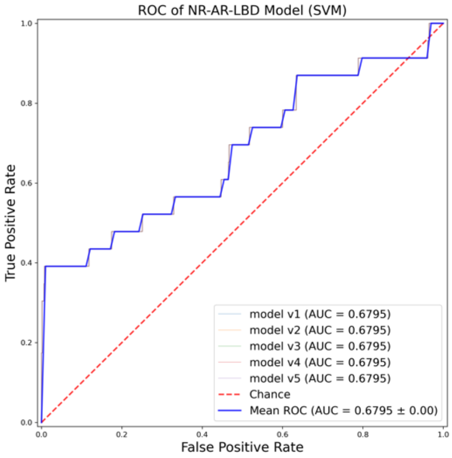

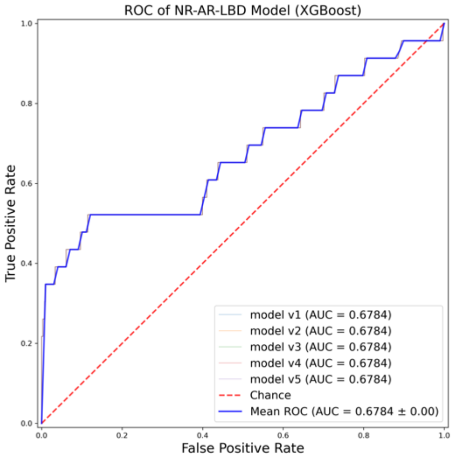

CM Models - NR-AR

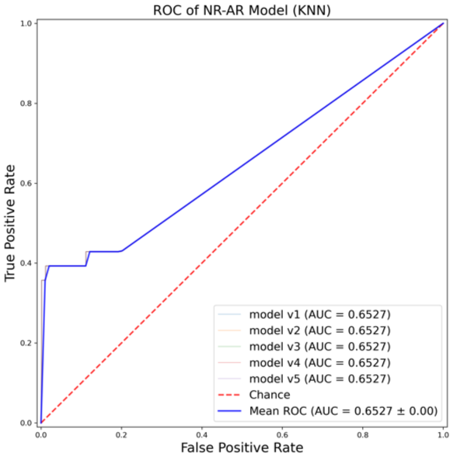

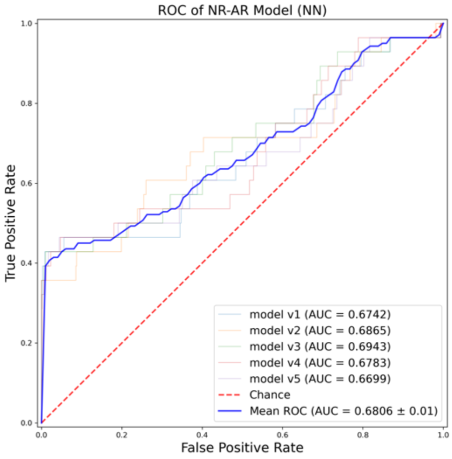

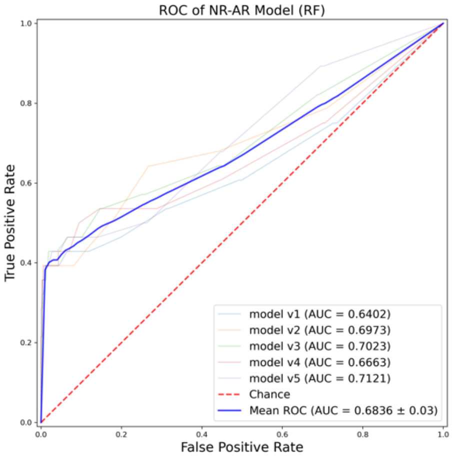

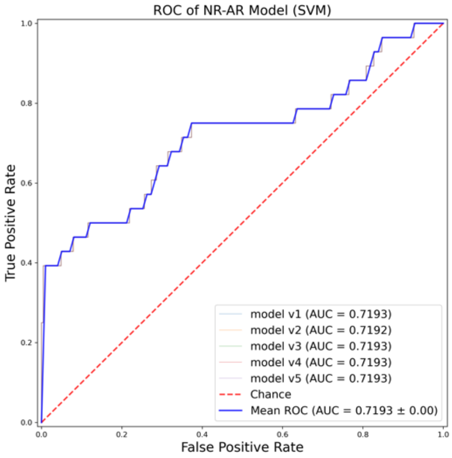

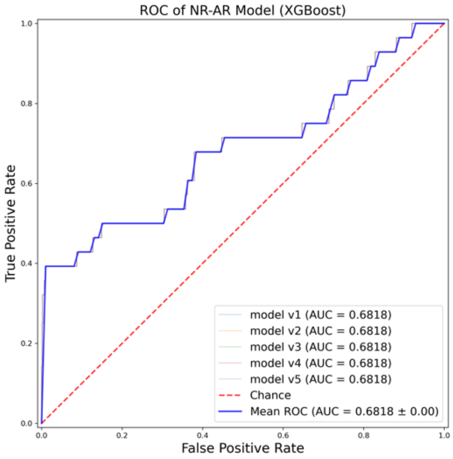

CM Models - NR-Aromatase

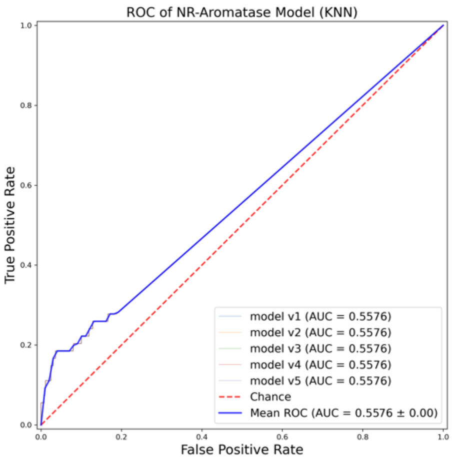

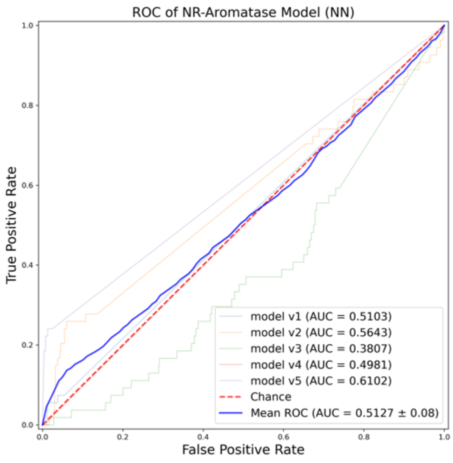

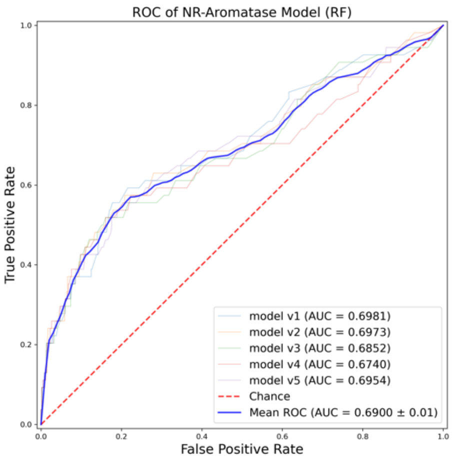

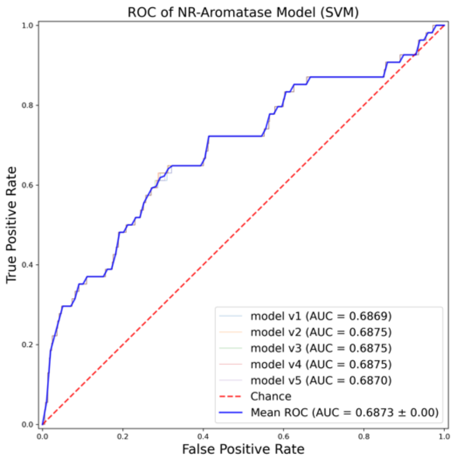

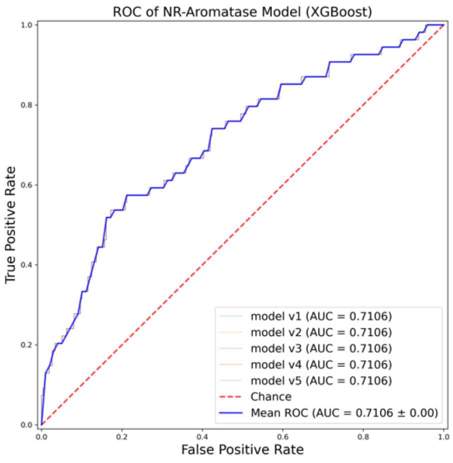

CM Models - NR-ER-LBD

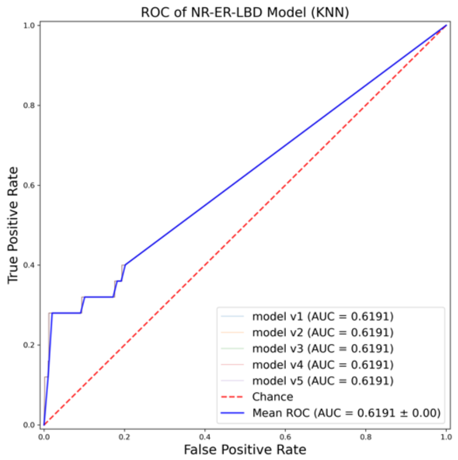

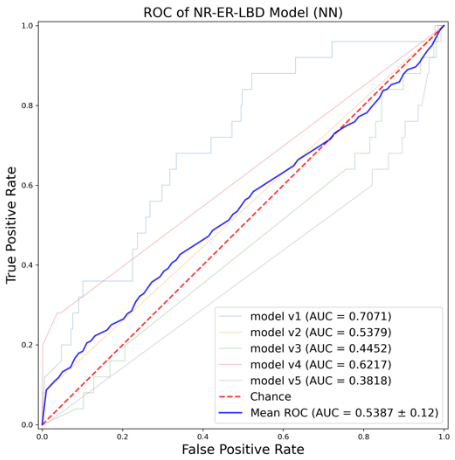

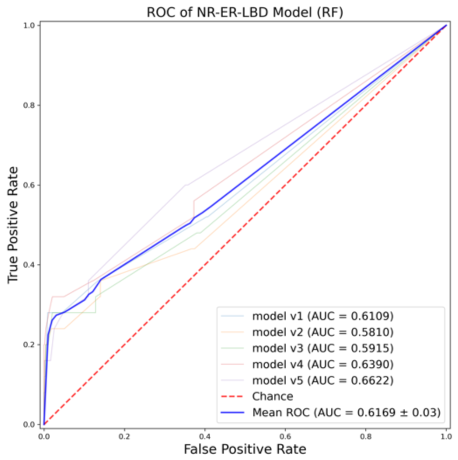

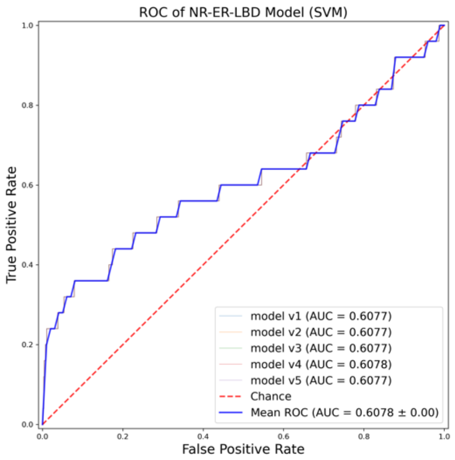

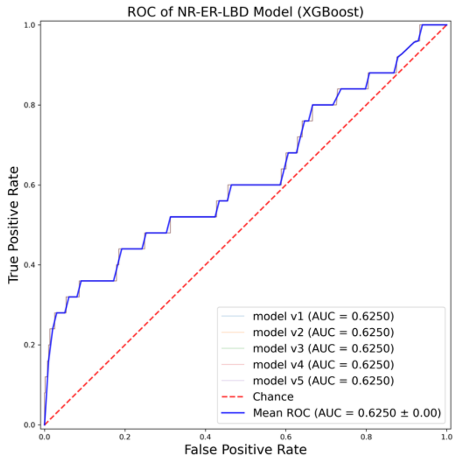

CM Models - NR-ER

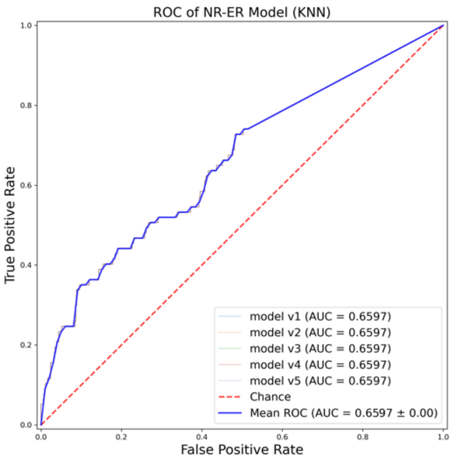

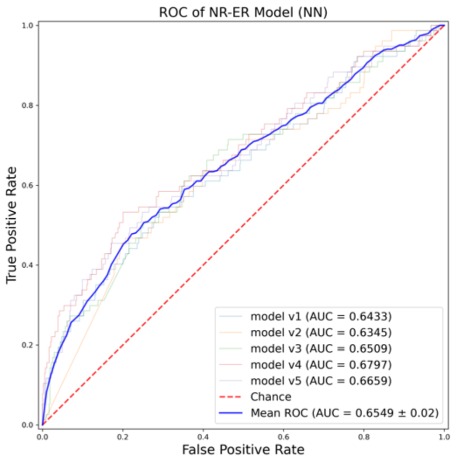

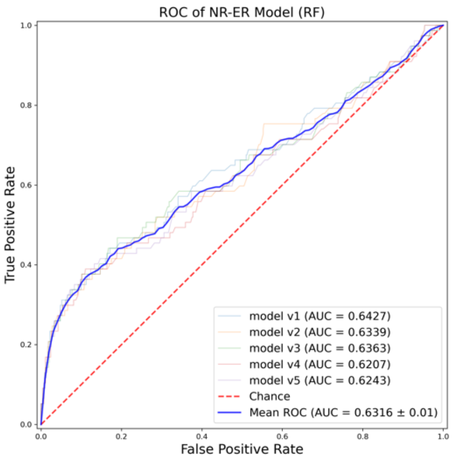

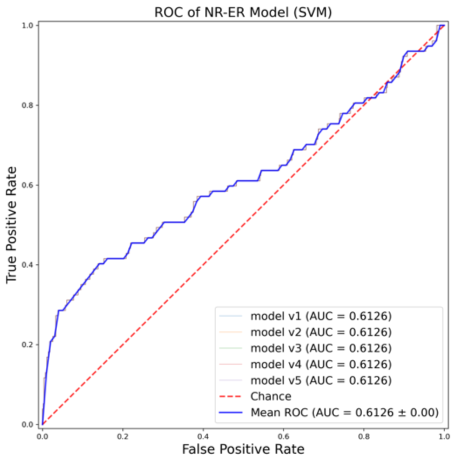

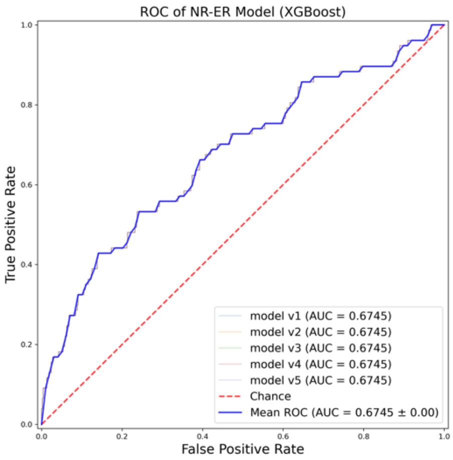

CM Models - NR-PPAR-gamma

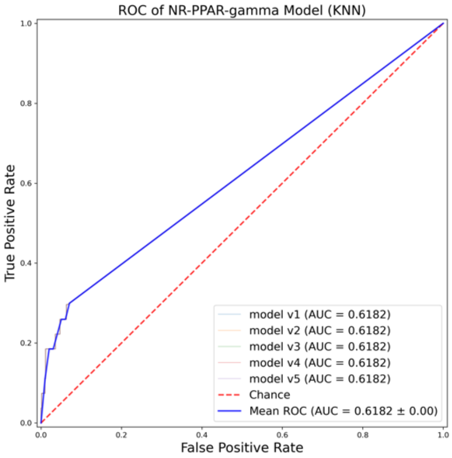

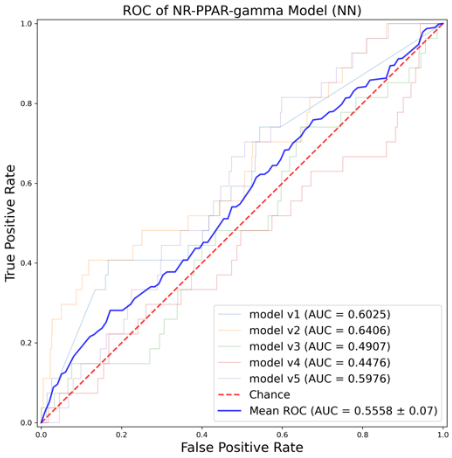

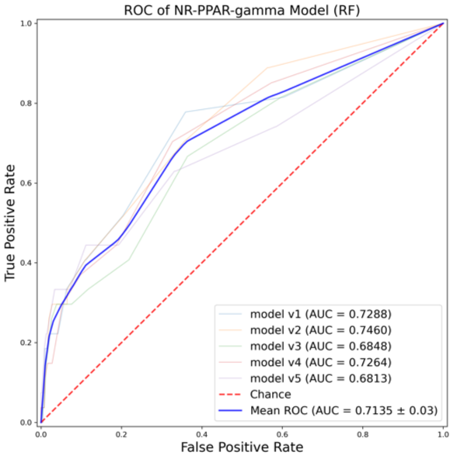

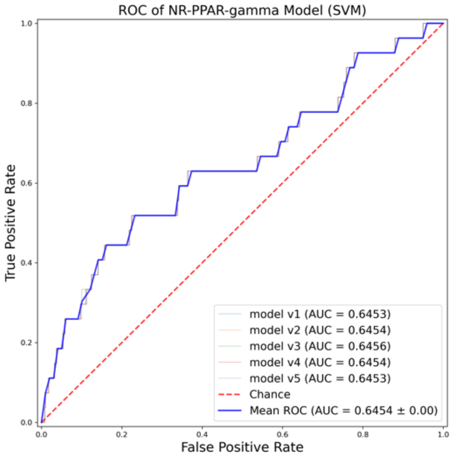

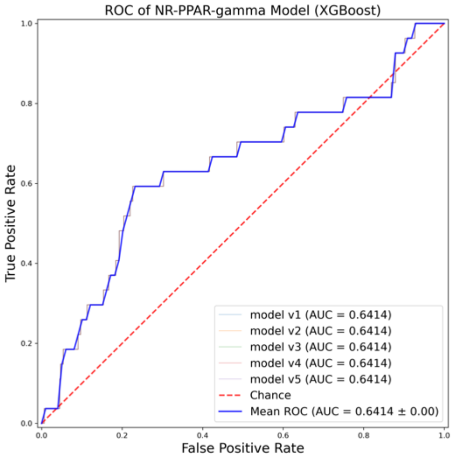

CM Models - SR-ARE

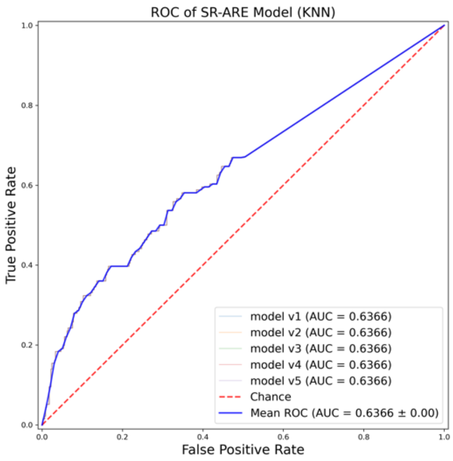

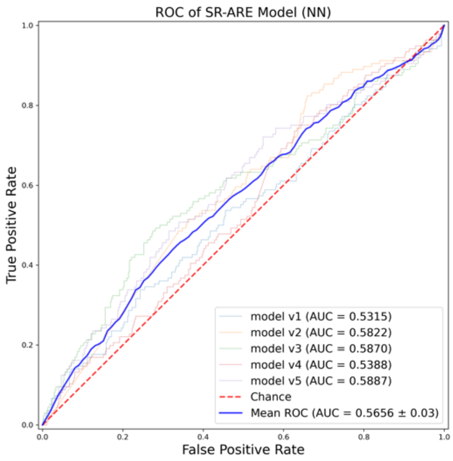

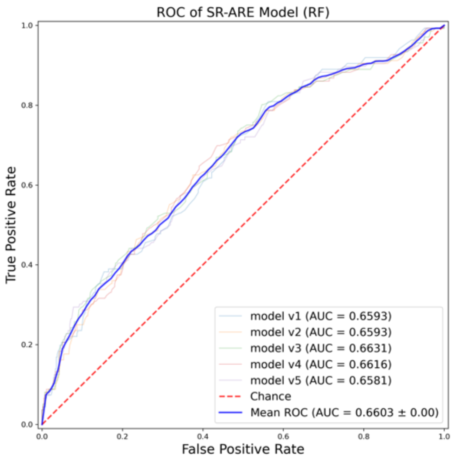

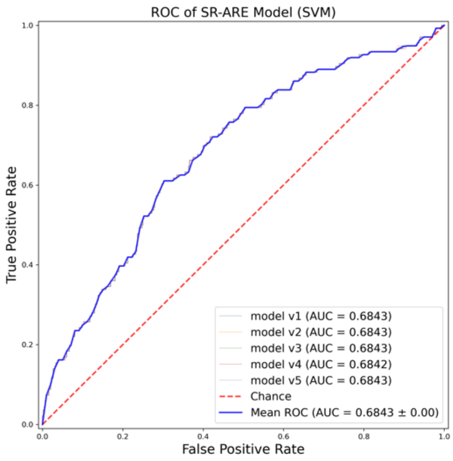

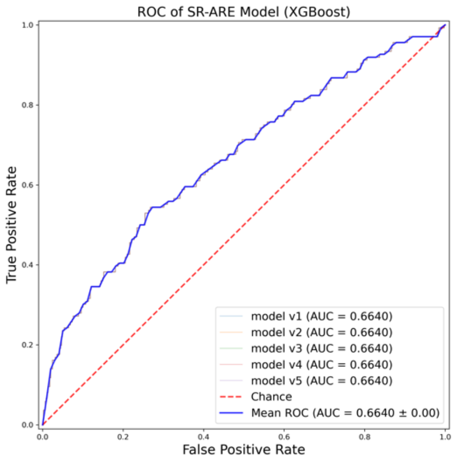

CM Models - SR-ATAD5

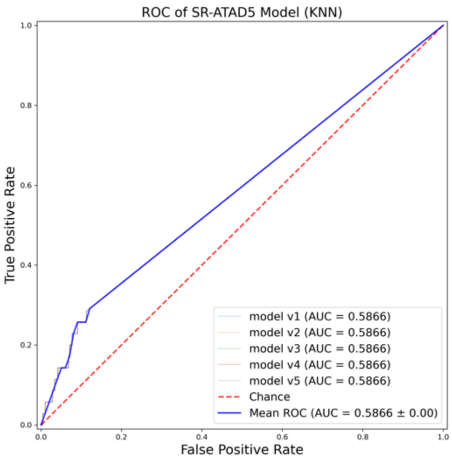

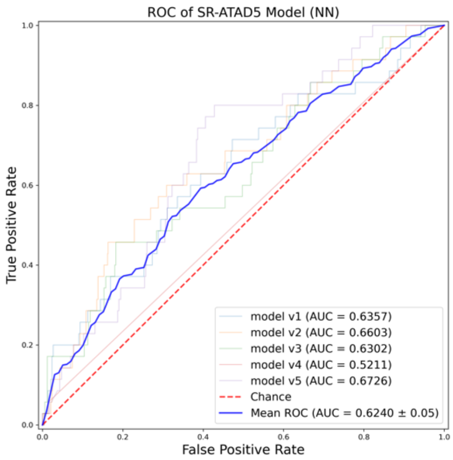

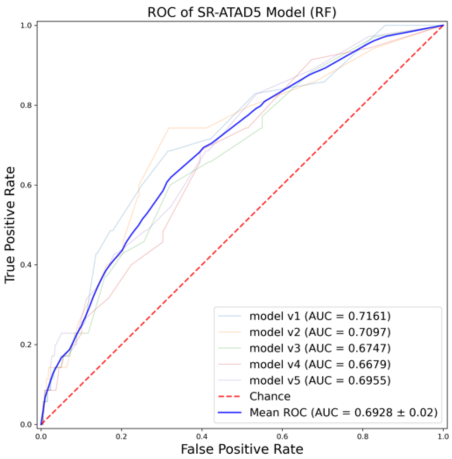

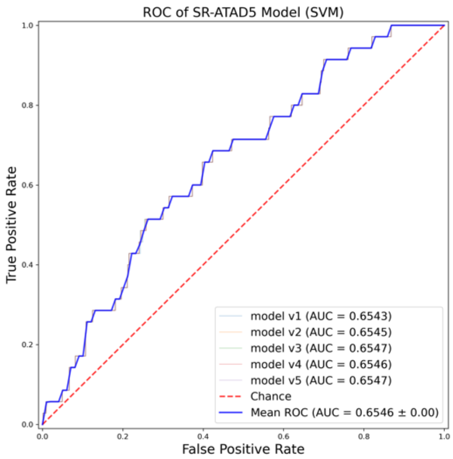

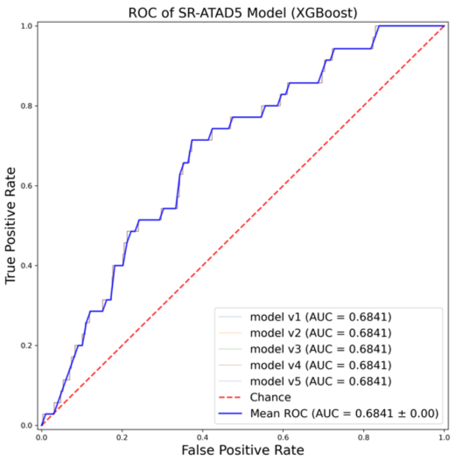

CM Models - SR-HSE

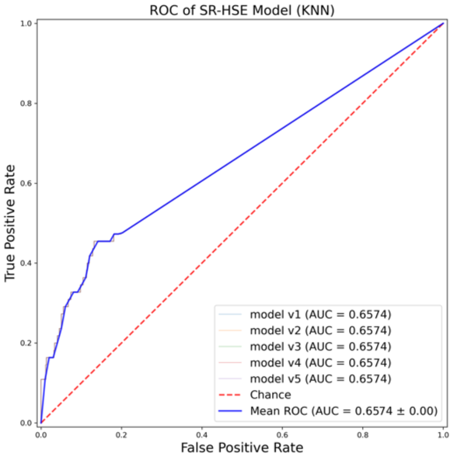

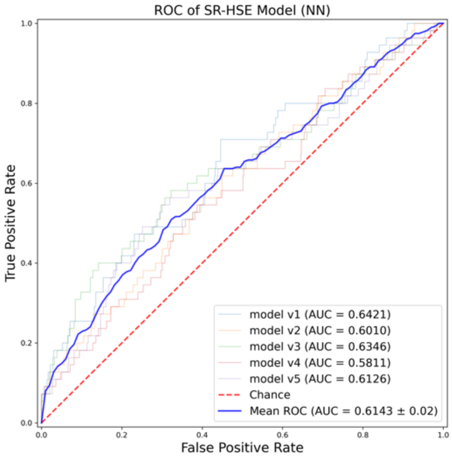

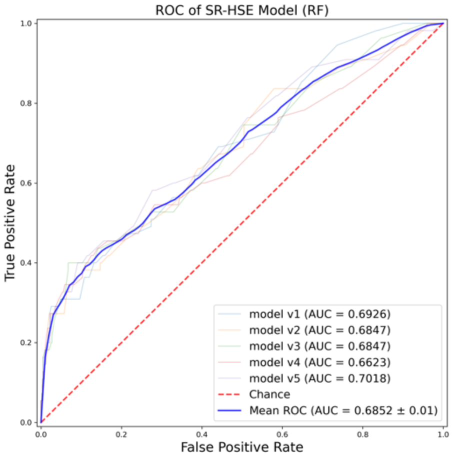

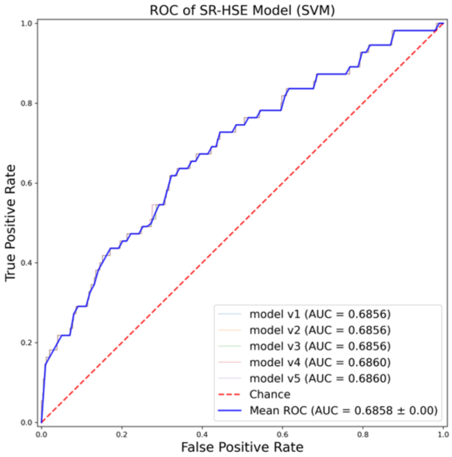

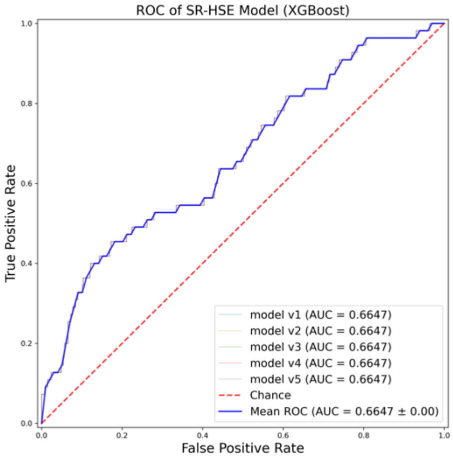

CM Models - SR-MMP

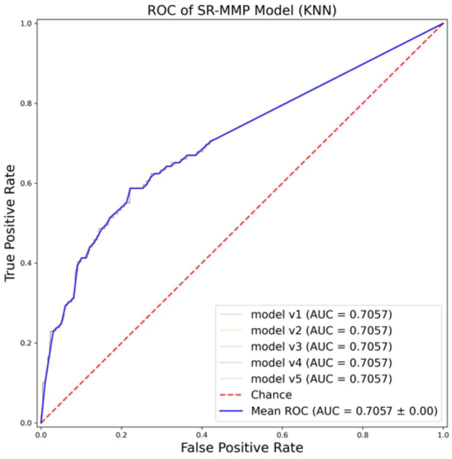

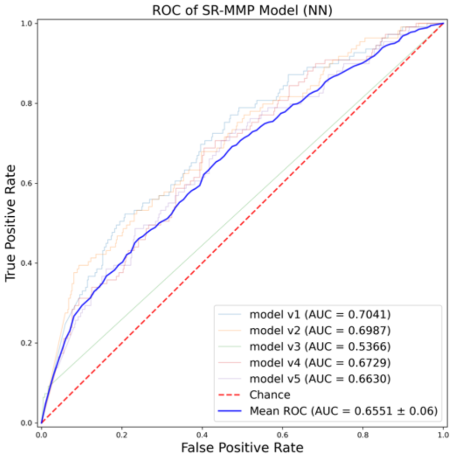

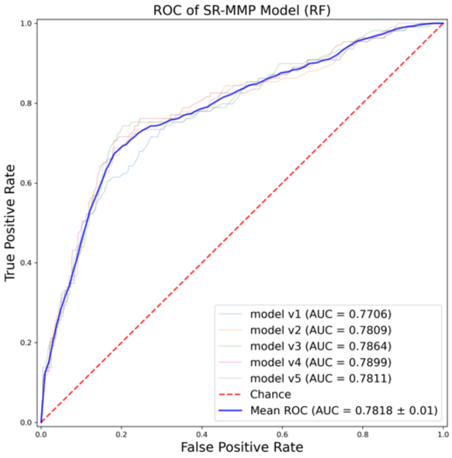

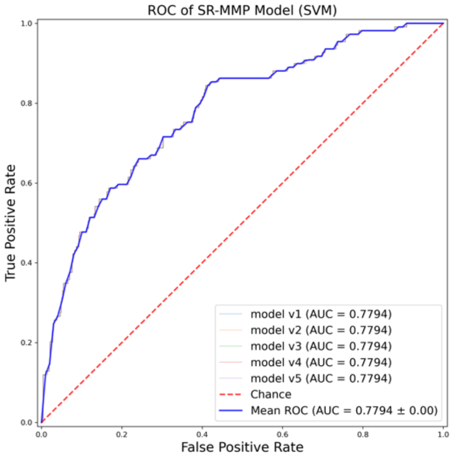

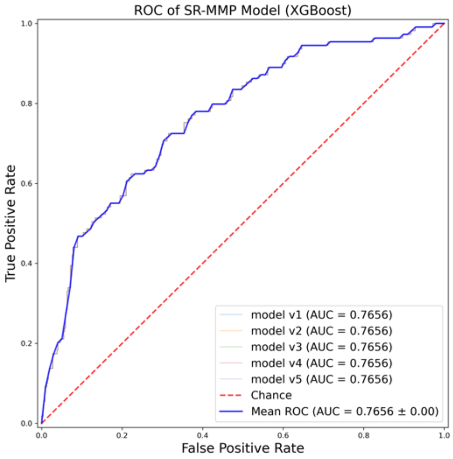

CM Models - SR-p53

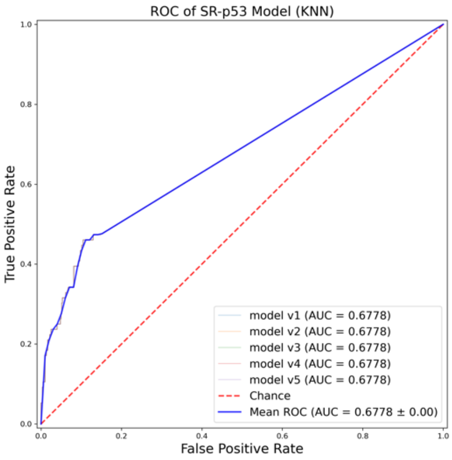

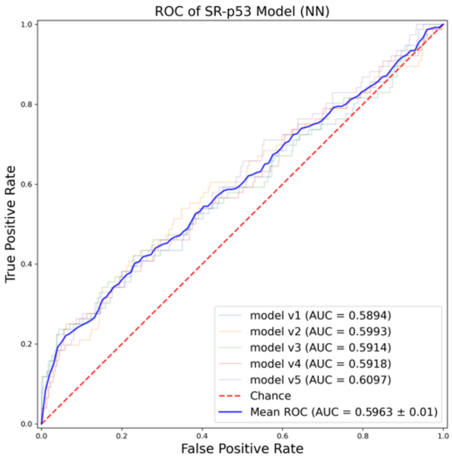

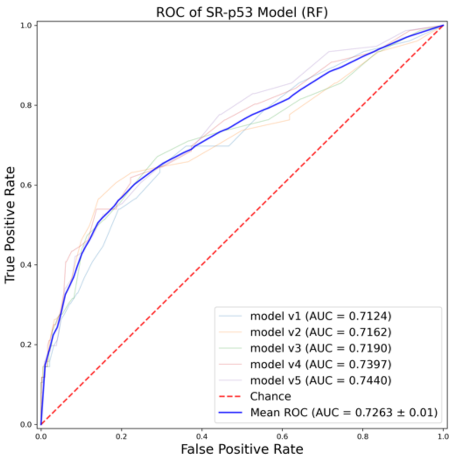

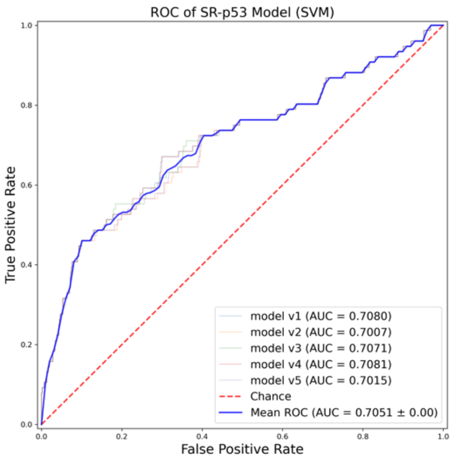

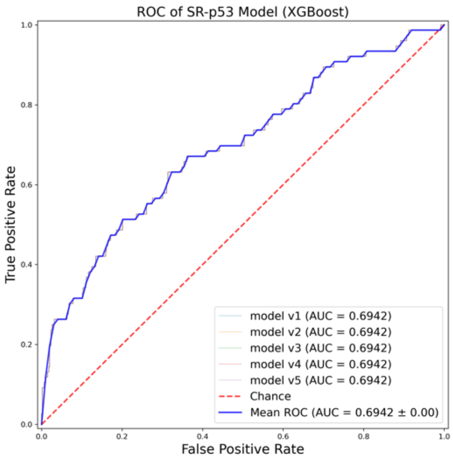

Figure S2. Details of the ROC curves of the SL-GCN models in 5 repeated experiments on the 12 prediction tasks.

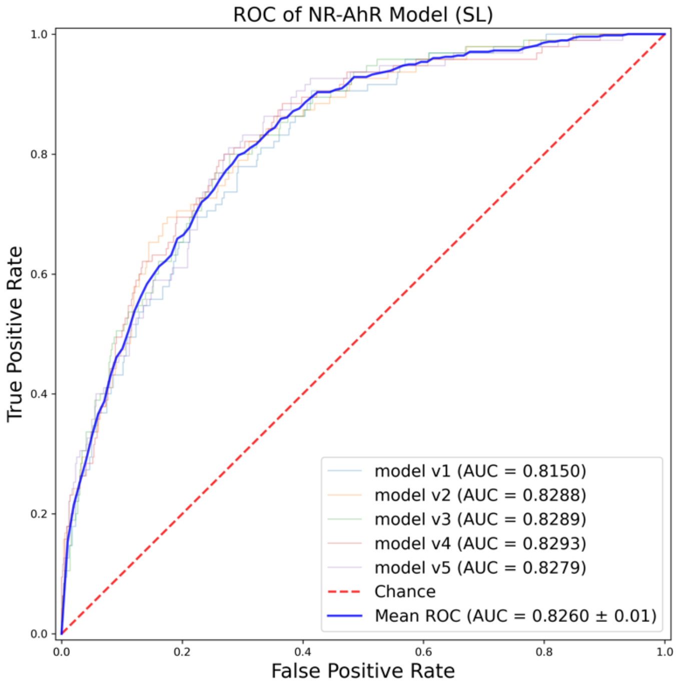

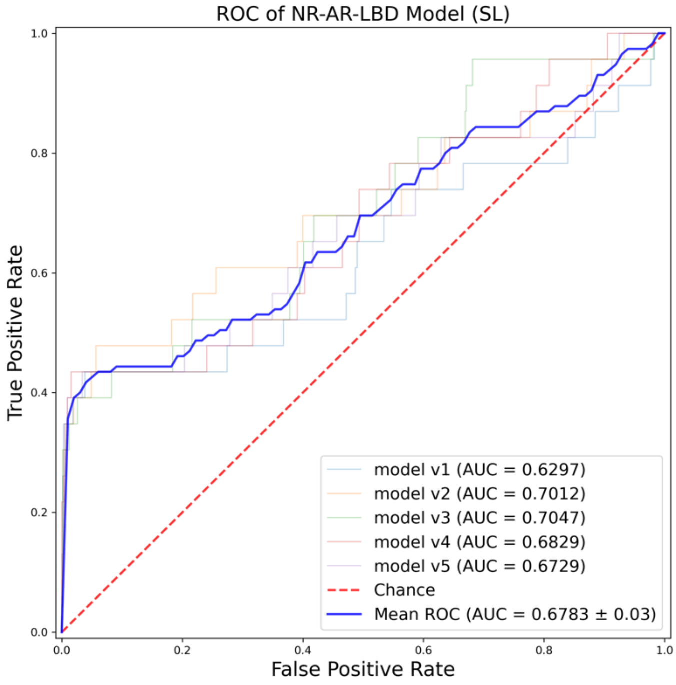

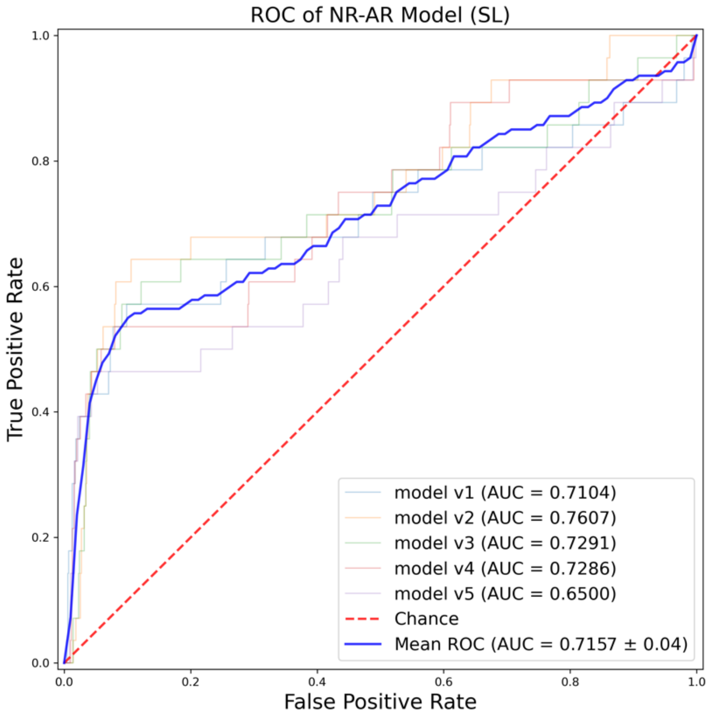

ROC of NR-AR Model (SL)
True Positive Rate
False Positive Rate
0.0
0.2
0.4
0.6
0.8
1.0
model v1 (AUC = 0.7104)
model v2 (AUC = 0.7607)
model v3 (AUC = 0.7291)
model v4 (AUC = 0.7286)
model v5 (AUC = 0.6500)
Chance
Mean ROC (AUC = 0.7157 ± 0.04)

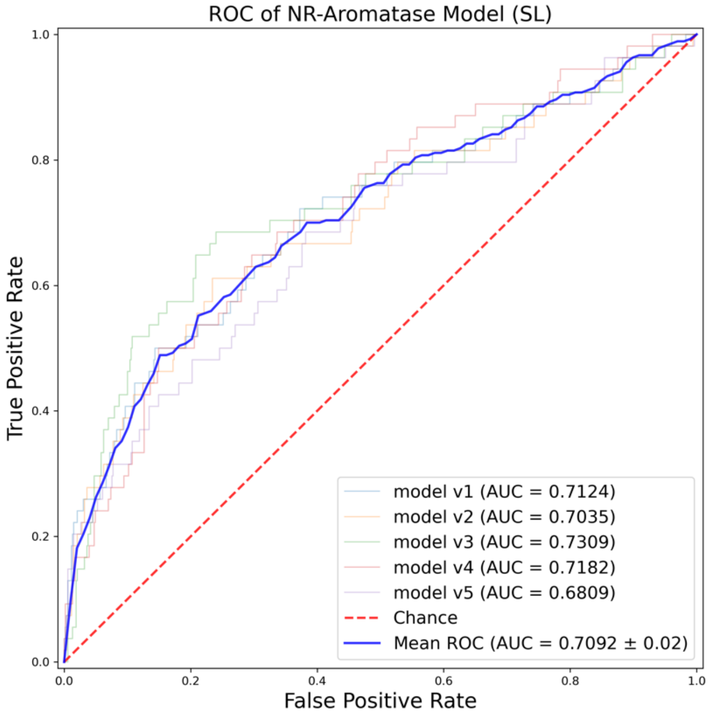

ROC of NR-Aromatase Model (SL)
True Positive Rate
False Positive Rate
0.0
0.2
0.4
0.6
0.8
1.0
model v1 (AUC = 0.7124)
model v2 (AUC = 0.7035)
model v3 (AUC = 0.7309)
model v4 (AUC = 0.7182)
model v5 (AUC = 0.6809)
Chance
Mean ROC (AUC = 0.7092 ± 0.02)

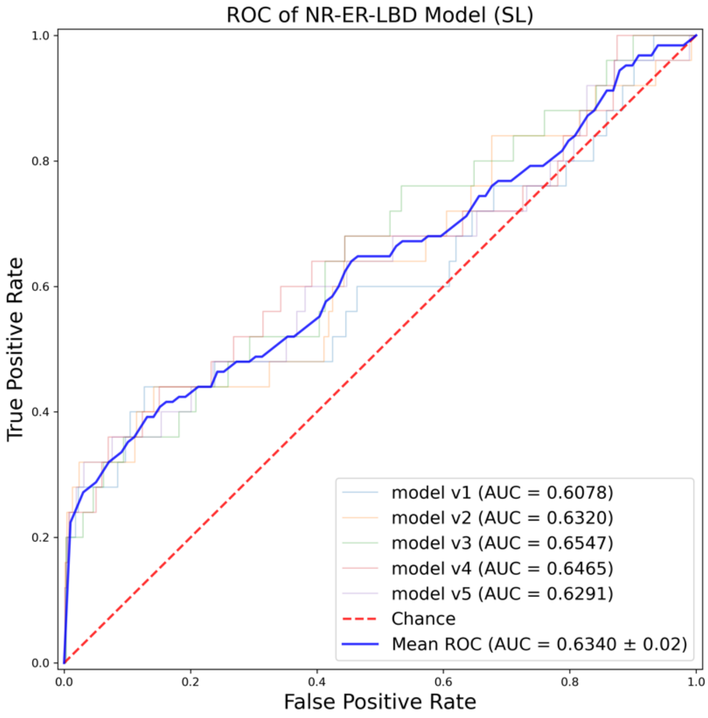
ROC of NR-ER-LBD Model (SL)
True Positive Rate
False Positive Rate
0.0
0.2
0.4
0.6
0.8
1.0
model v1 (AUC = 0.6078)
model v2 (AUC = 0.6320)
model v3 (AUC = 0.6547)
model v4 (AUC = 0.6465)
model v5 (AUC = 0.6291)
Chance
Mean ROC (AUC = 0.6340 ± 0.02)

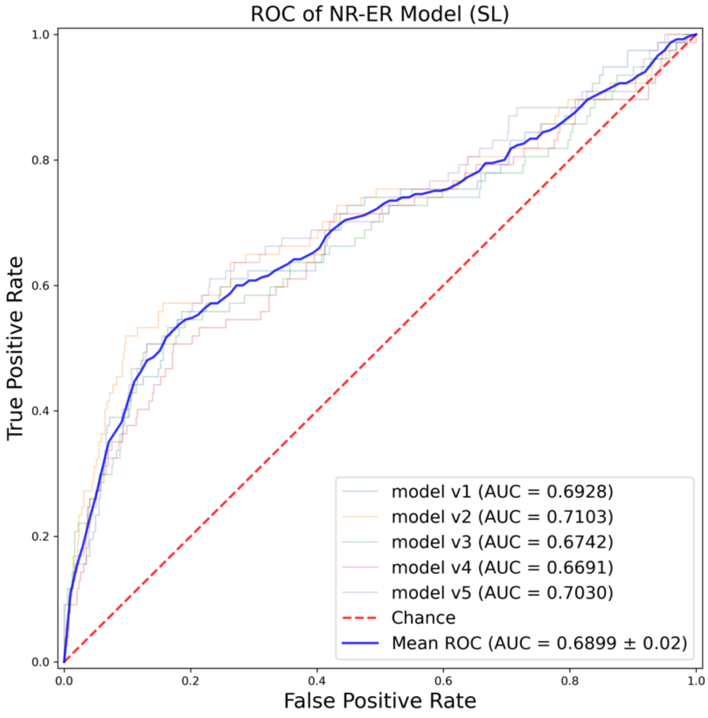
ROC of NR-ER Model (SL)
True Positive Rate
False Positive Rate
0.0
0.2
0.4
0.6
0.8
1.0
model v1 (AUC = 0.6928)
model v2 (AUC = 0.7103)
model v3 (AUC = 0.6742)
model v4 (AUC = 0.6691)
model v5 (AUC = 0.7030)
Chance
Mean ROC (AUC = 0.6899 ± 0.02)

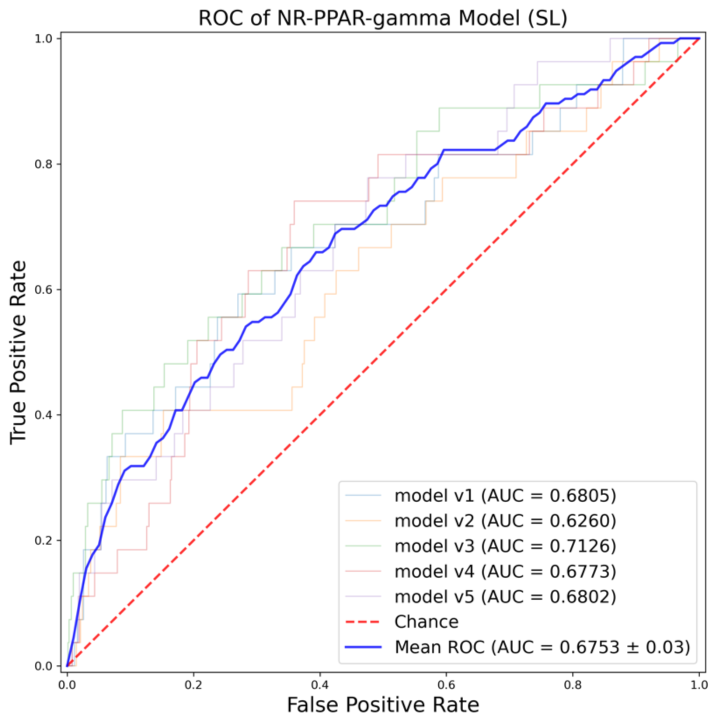
ROC of NR-PPAR-gamma Model (SL)
True Positive Rate
False Positive Rate
0.0
0.2
0.4
0.6
0.8
1.0
model v1 (AUC = 0.6805)
model v2 (AUC = 0.6260)
model v3 (AUC = 0.7126)
model v4 (AUC = 0.6773)
model v5 (AUC = 0.6802)
Chance
Mean ROC (AUC = 0.6753 ± 0.03)

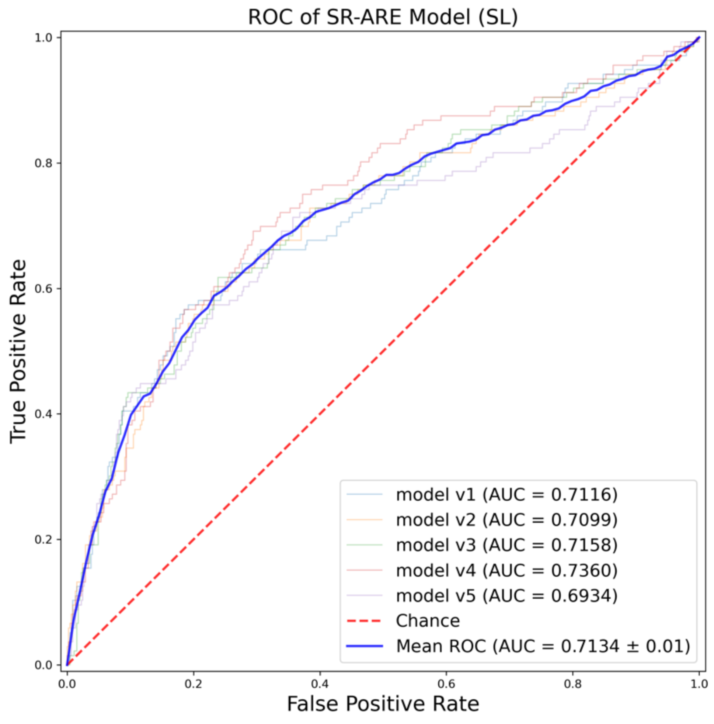
ROC of SR-ARE Model (SL)
True Positive Rate
False Positive Rate
0.0
0.2
0.4
0.6
0.8
1.0
model v1 (AUC = 0.7116)
model v2 (AUC = 0.7099)
model v3 (AUC = 0.7158)
model v4 (AUC = 0.7360)
model v5 (AUC = 0.6934)
Chance
Mean ROC (AUC = 0.7134 ± 0.01)

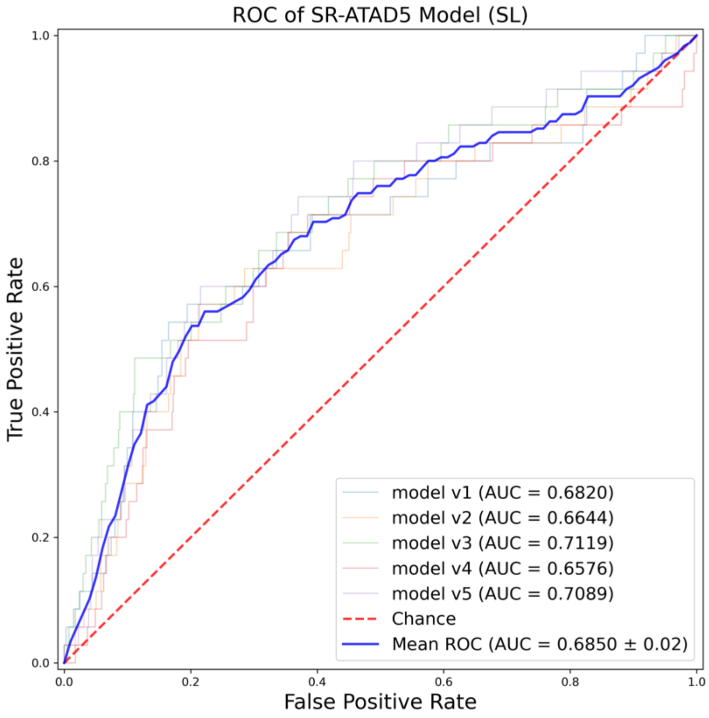
ROC of SR-ATAD5 Model (SL)
True Positive Rate
False Positive Rate
0.0
0.2
0.4
0.6
0.8
1.0
model v1 (AUC = 0.6820)
model v2 (AUC = 0.6644)
model v3 (AUC = 0.7119)
model v4 (AUC = 0.6576)
model v5 (AUC = 0.7089)
Chance
Mean ROC (AUC = 0.6850 ± 0.02)

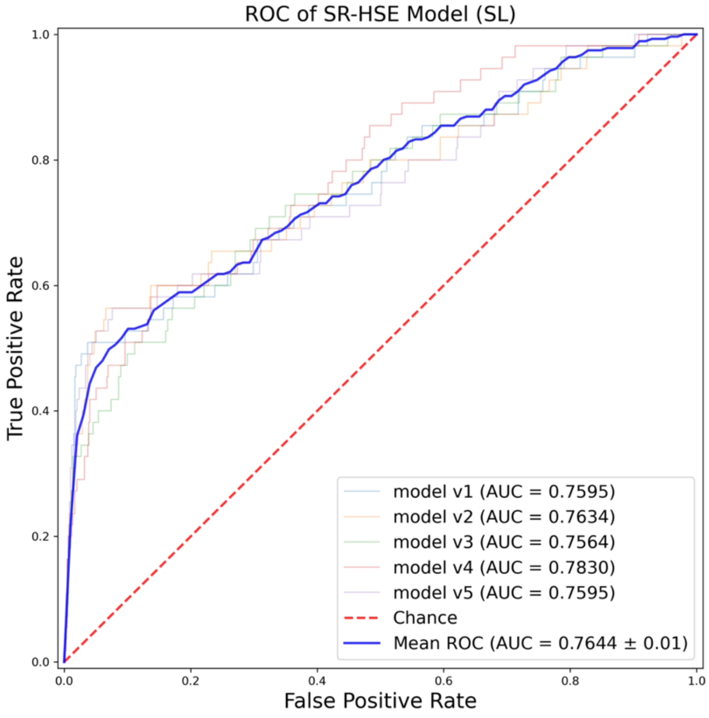
ROC of SR-HSE Model (SL)
True Positive Rate
False Positive Rate
0.0
0.2
0.4
0.6
0.8
1.0
model v1 (AUC = 0.7595)
model v2 (AUC = 0.7634)
model v3 (AUC = 0.7564)
model v4 (AUC = 0.7830)
model v5 (AUC = 0.7595)
Chance
Mean ROC (AUC = 0.7644 ± 0.01)

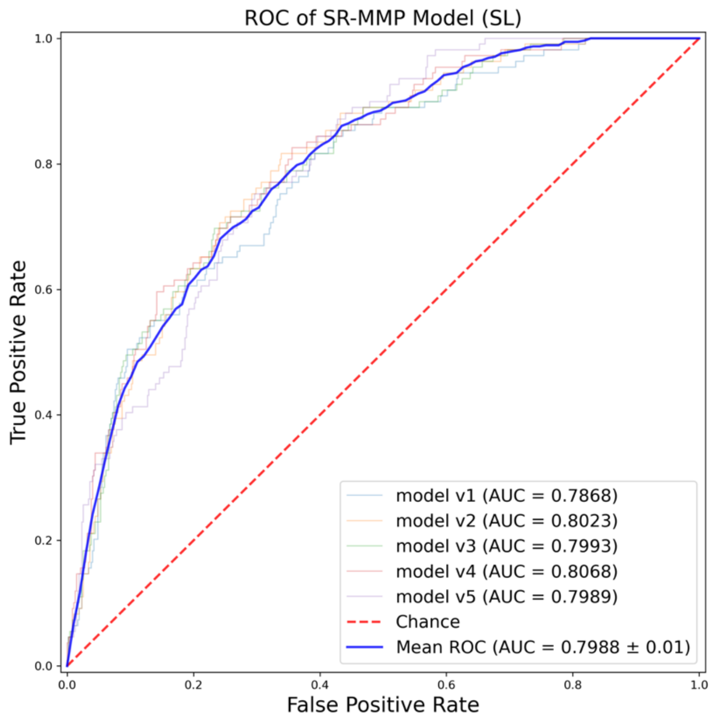

ROC of SR-MMP Model (SL)
True Positive Rate
False Positive Rate
0.0
0.2
0.4
0.6
0.8
1.0
model v1 (AUC = 0.7868)
model v2 (AUC = 0.8023)
model v3 (AUC = 0.7993)
model v4 (AUC = 0.8068)
model v5 (AUC = 0.7989)
Chance
Mean ROC (AUC = 0.7988 ± 0.01)

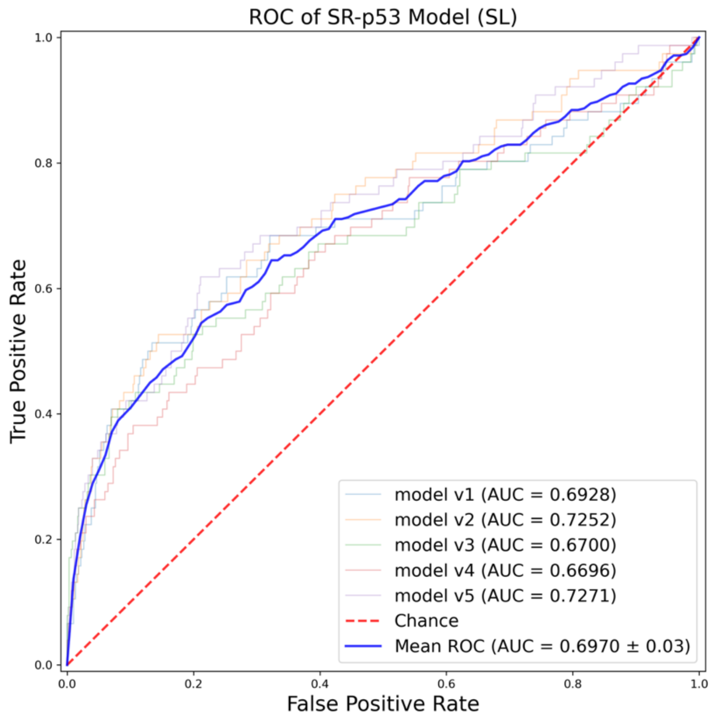

ROC of SR-p53 Model (SL)
True Positive Rate
False Positive Rate
0.0
0.2
0.4
0.6
0.8
1.0
model v1 (AUC = 0.6928)
model v2 (AUC = 0.7252)
model v3 (AUC = 0.6700)
model v4 (AUC = 0.6696)
model v5 (AUC = 0.7271)
Chance
Mean ROC (AUC = 0.6970 ± 0.03)

Figure S3. Details of the ROC curves of the SL-GCN models with different unlabeled ratios (R_u) in 5 repeated experiments on the 12 prediction tasks.

SSL-GCN Models - NR-AhR

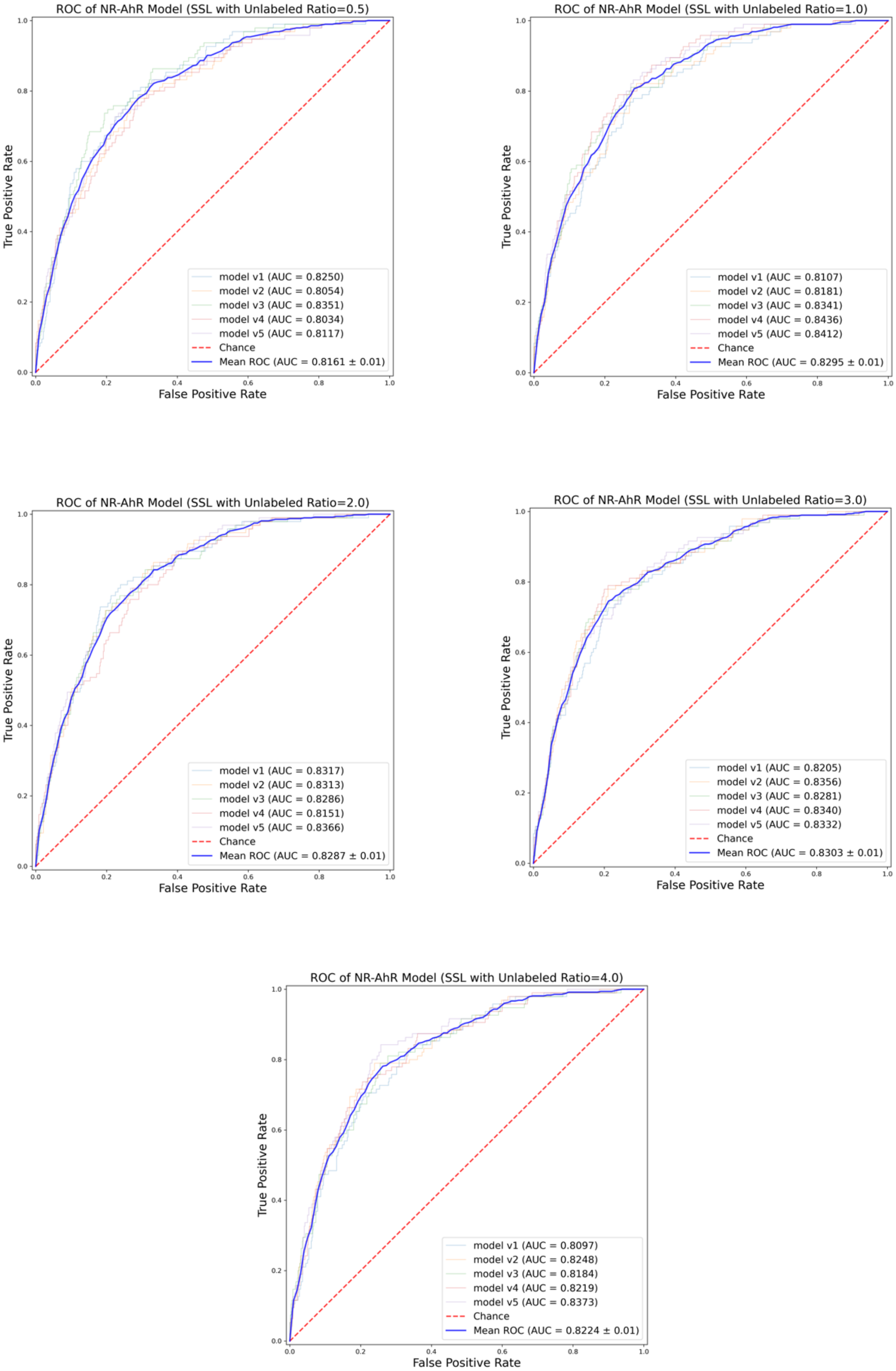

SSL-GCN Models - NR-AR-LBD

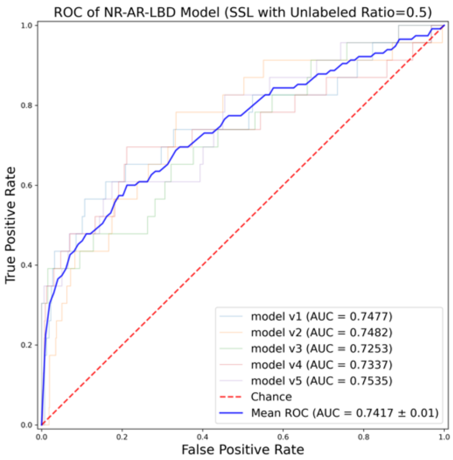

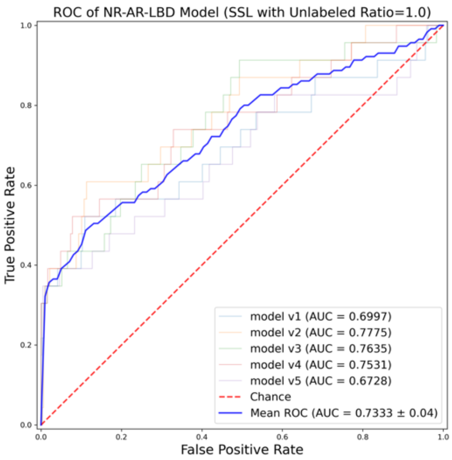

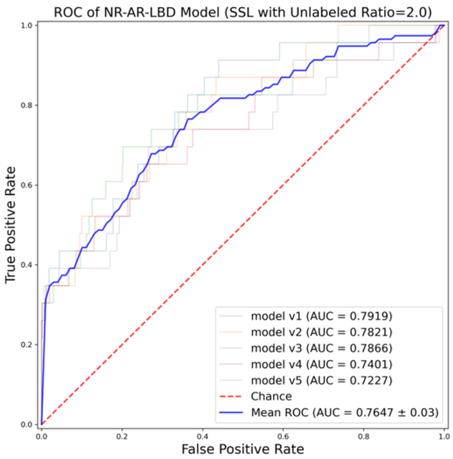

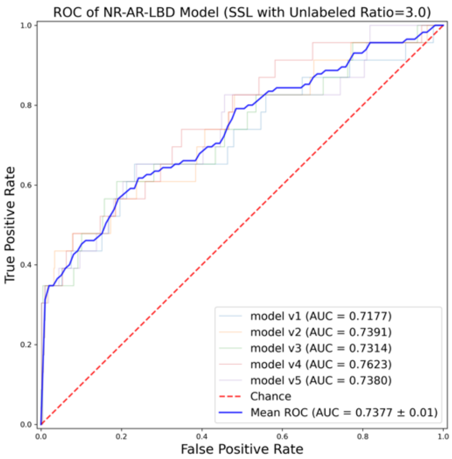

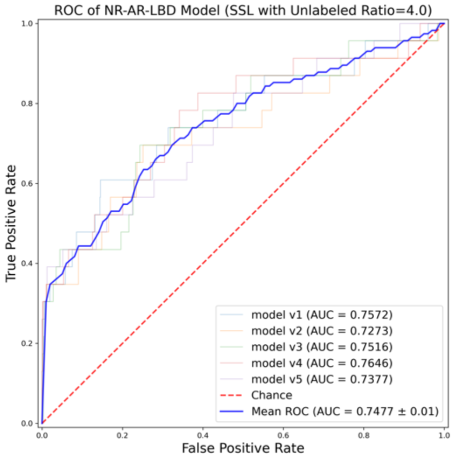

SSL-GCN Models - NR-AR

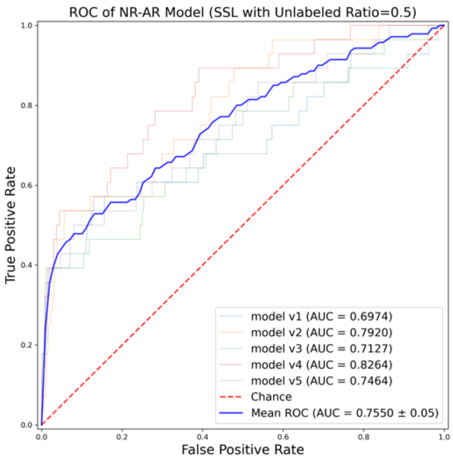

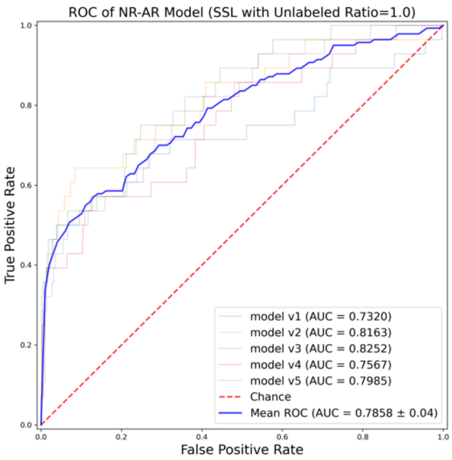

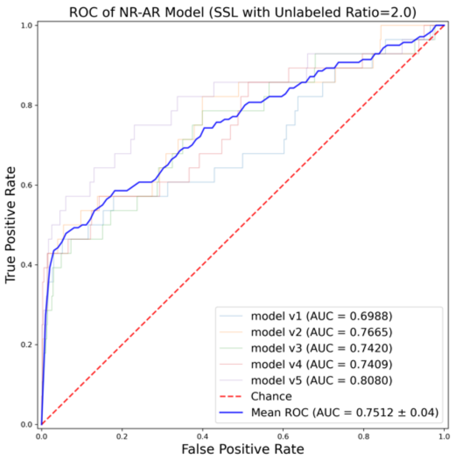

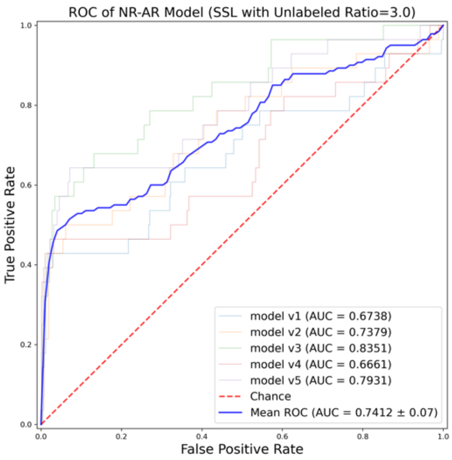

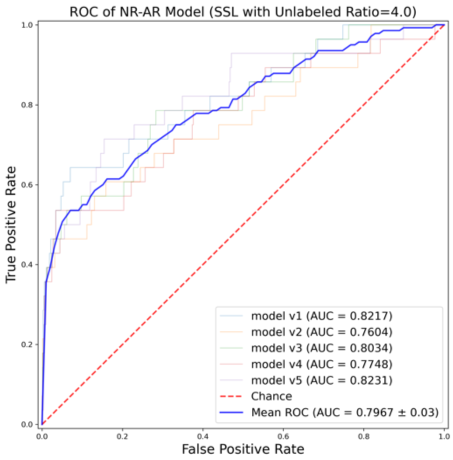

SSL-GCN Models - NR-Aromatase

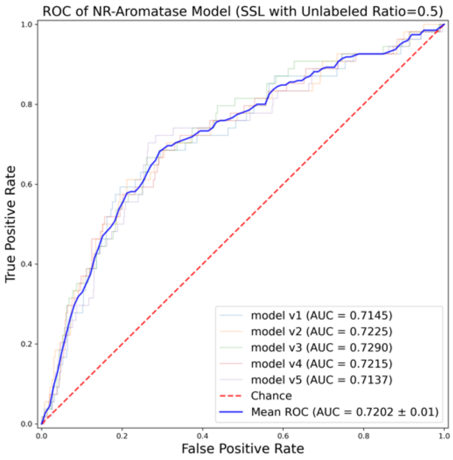

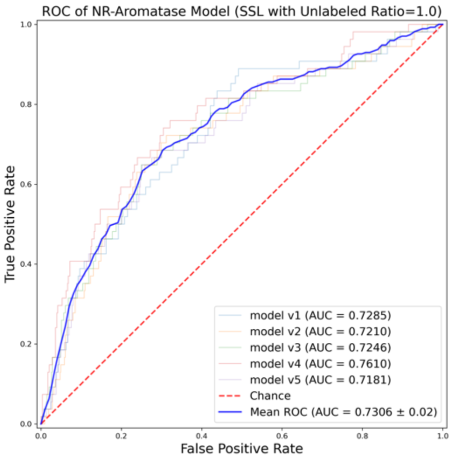

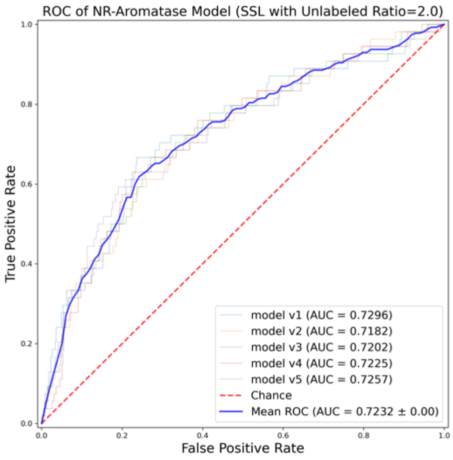

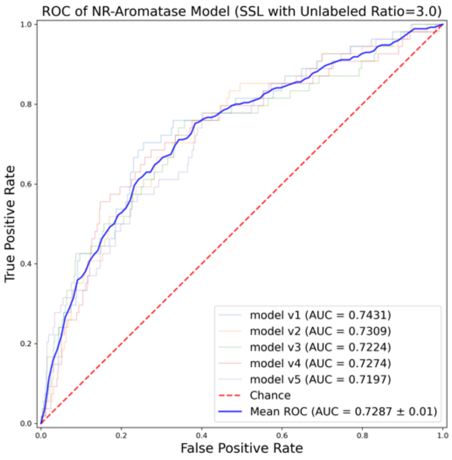

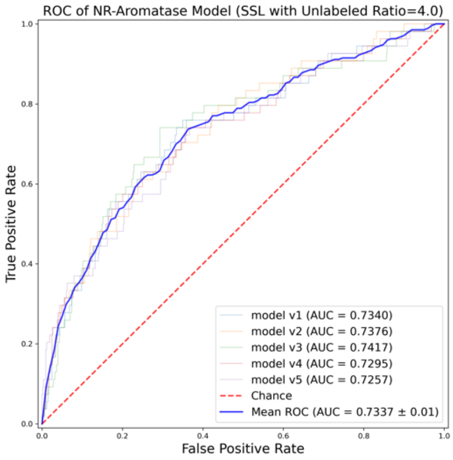

SSL-GCN Models - NR-ER-LBD

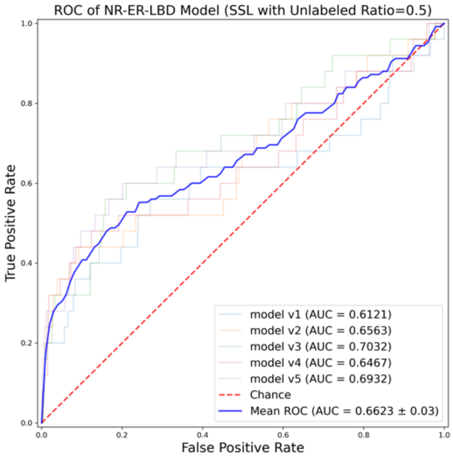

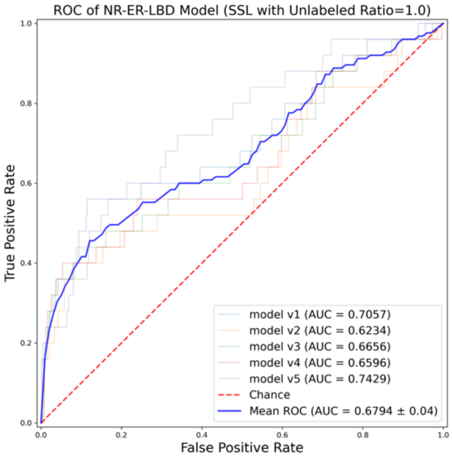

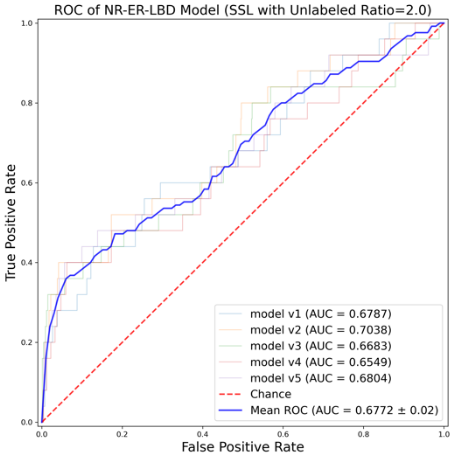

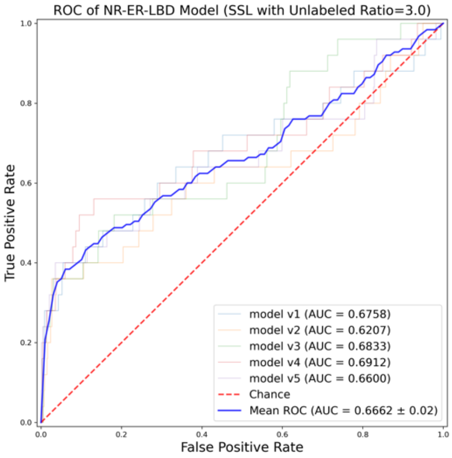

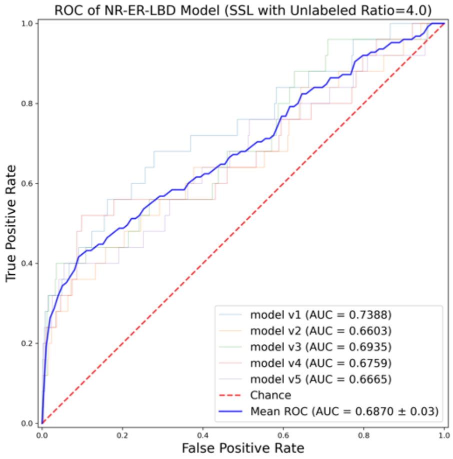

SSL-GCN Models - NR-ER

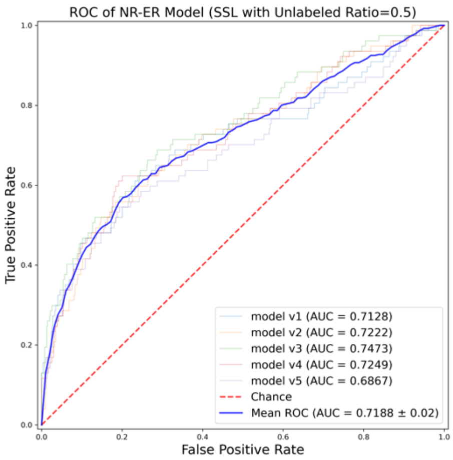

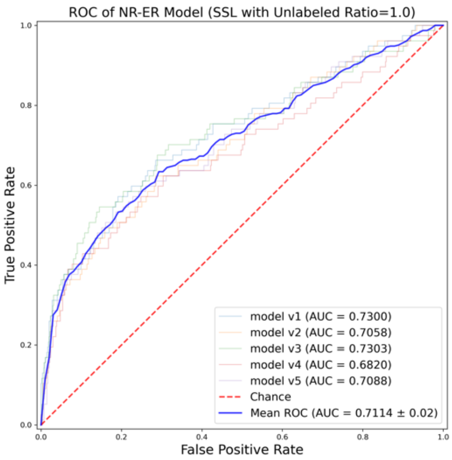

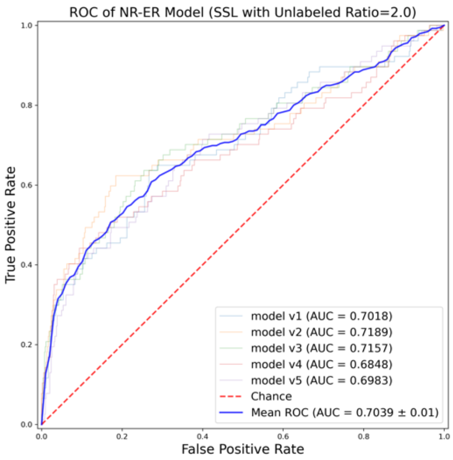

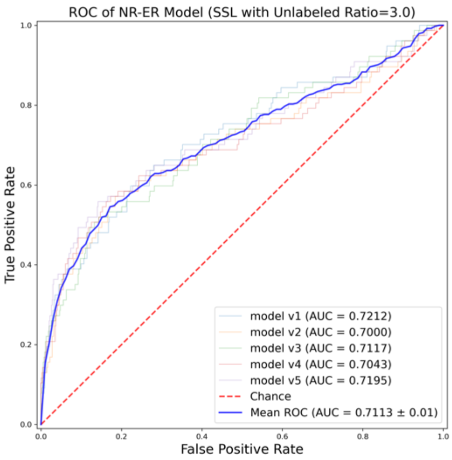

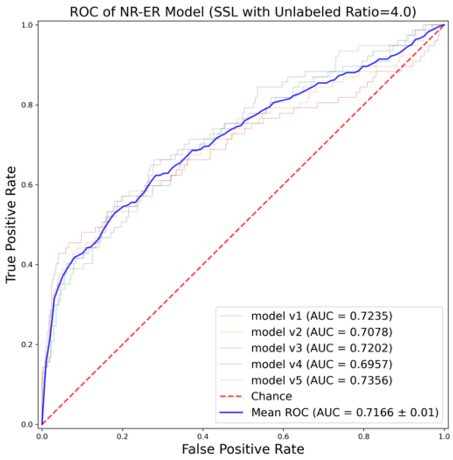

SSL-GCN Models - NR-PPAR-gamma

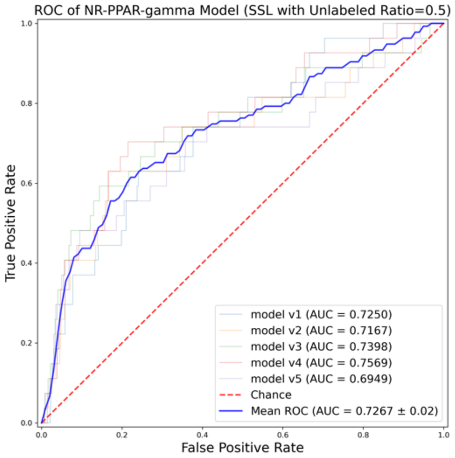

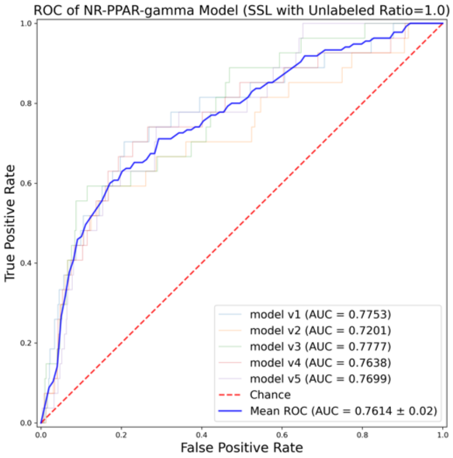

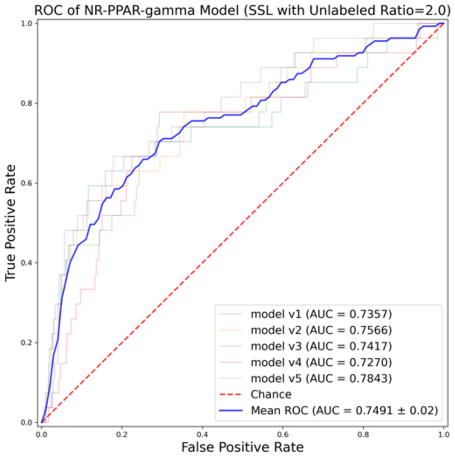

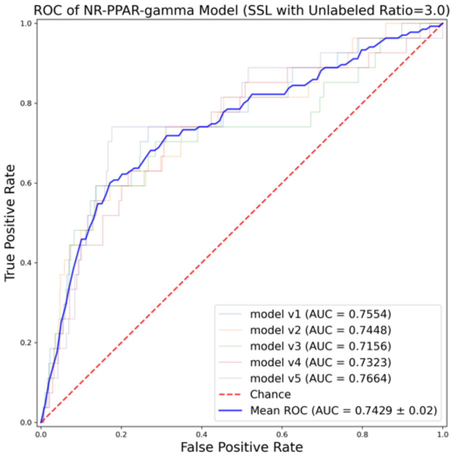

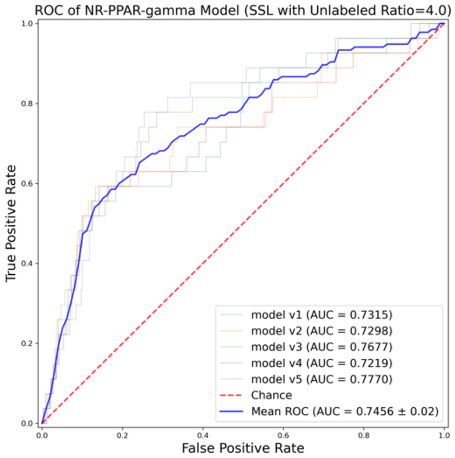

SSL-GCN Models - SR-ARE

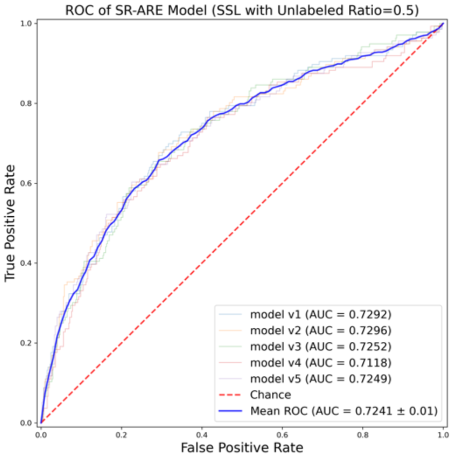

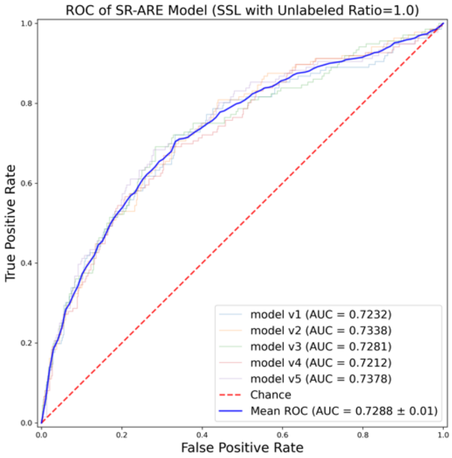

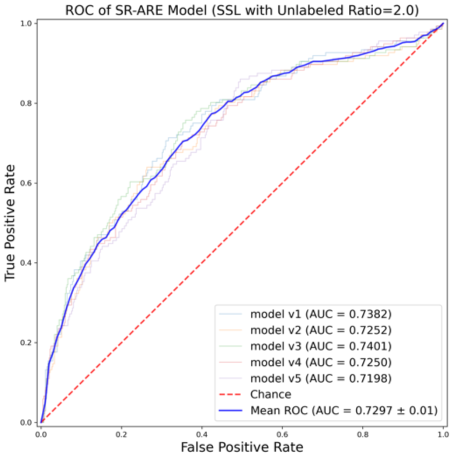

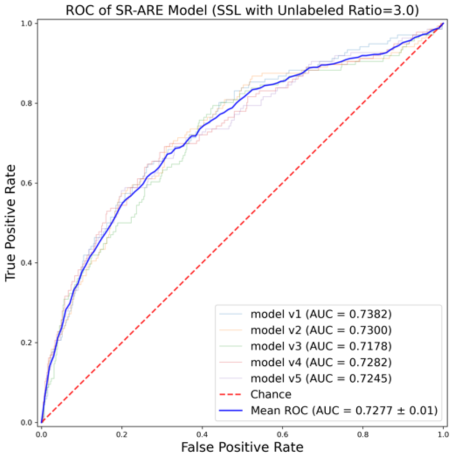

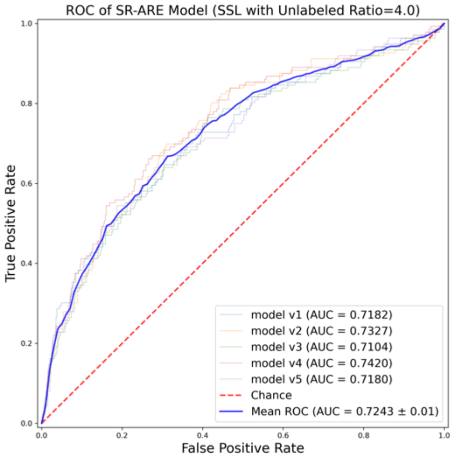

SSL-GCN Models - SR-ATAD5

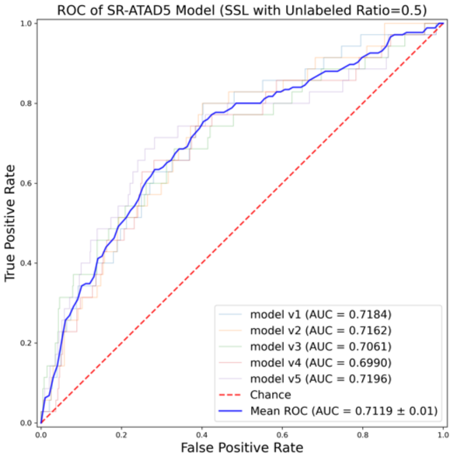

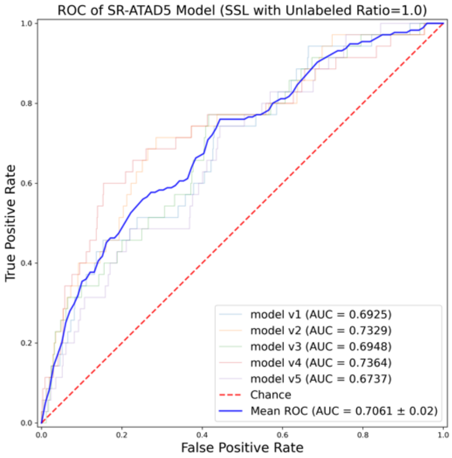

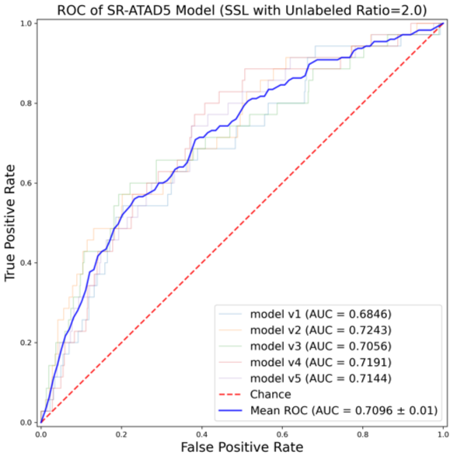

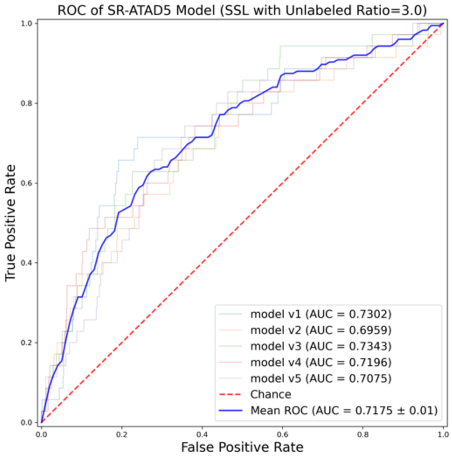

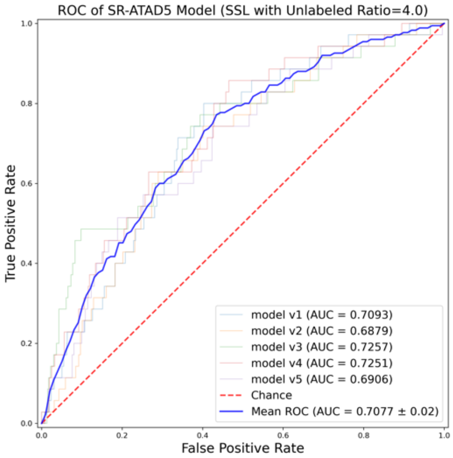

SSL-GCN Models - SR-HSE

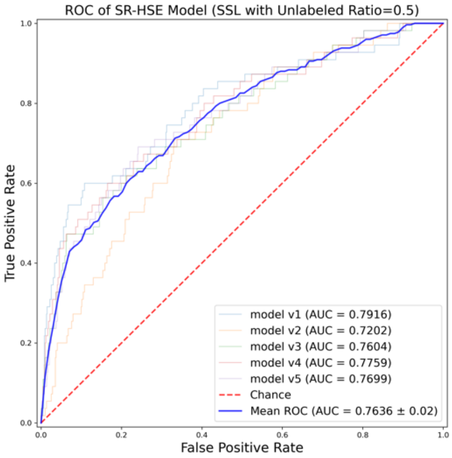

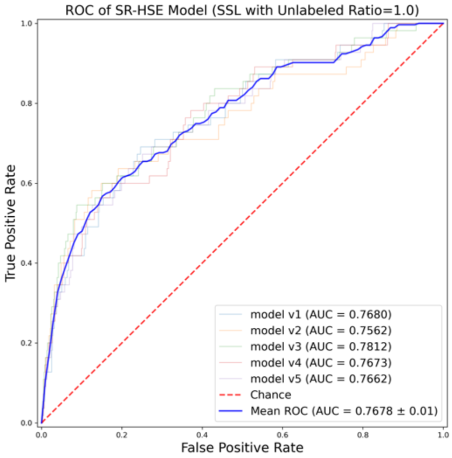

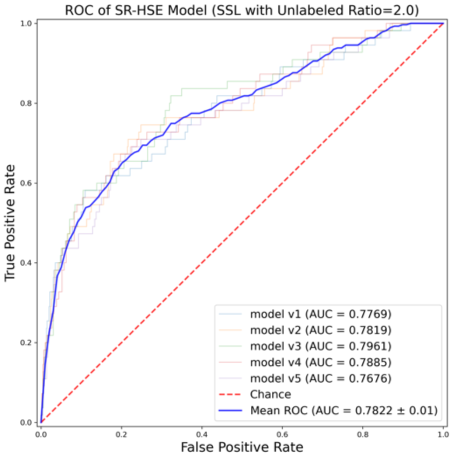

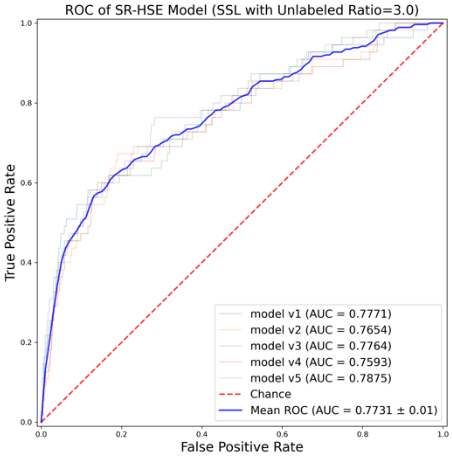

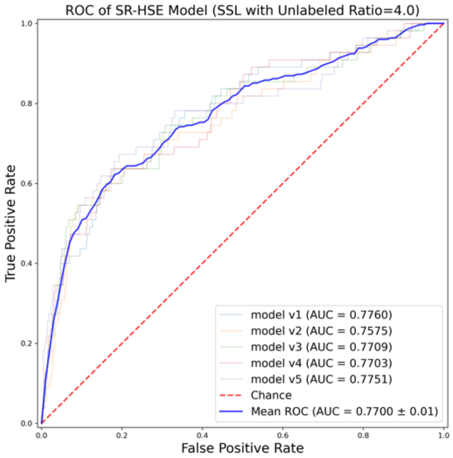

SSL-GCN Models - SR-MMP

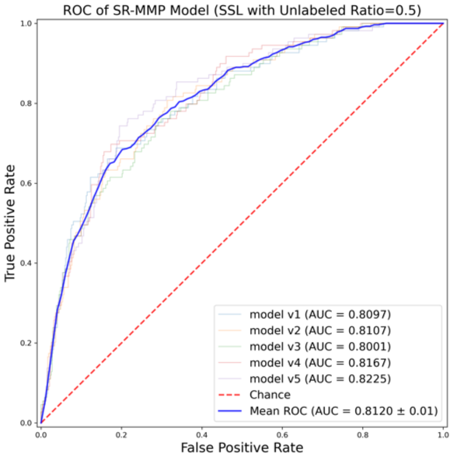

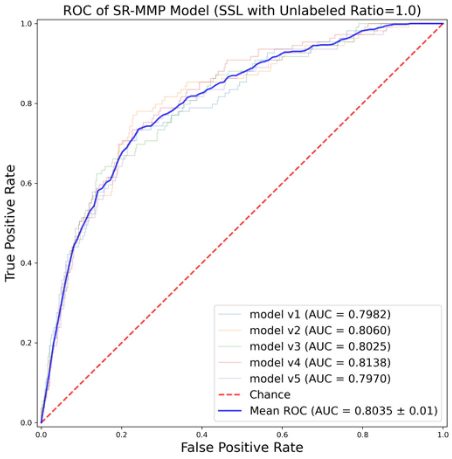

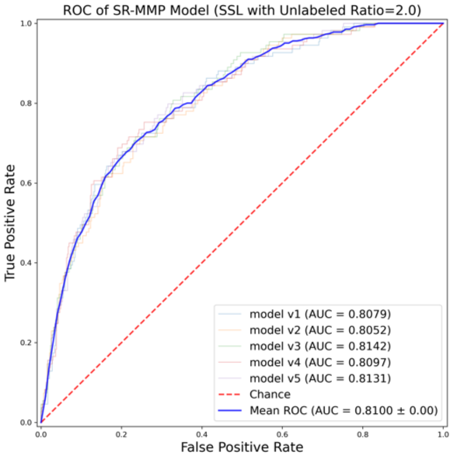

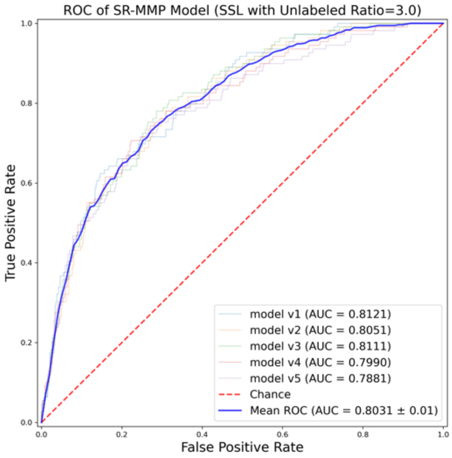

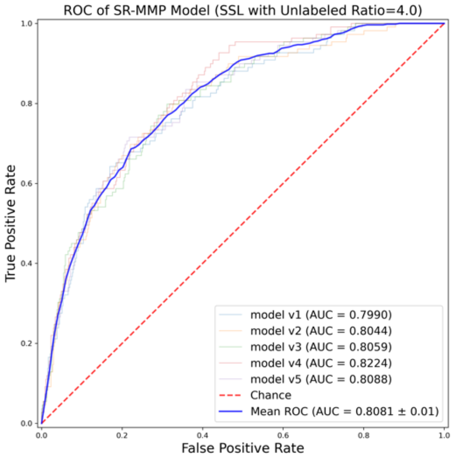

SSL-GCN Models - SR-p53

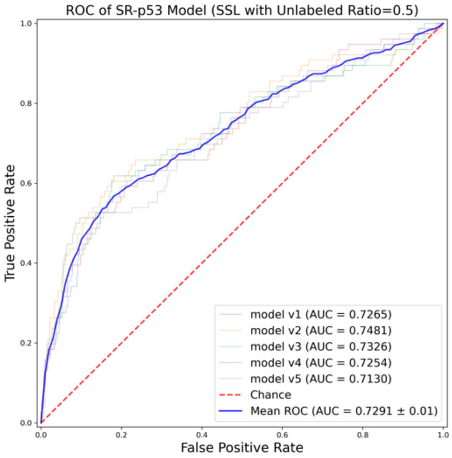

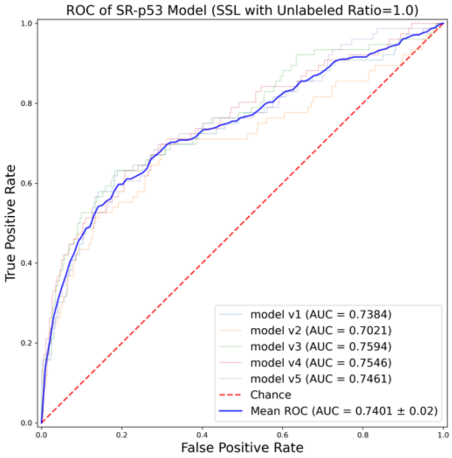

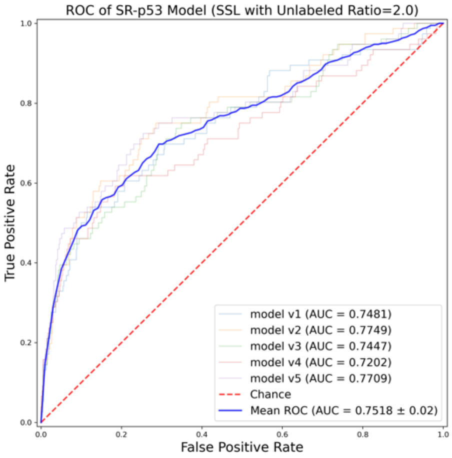

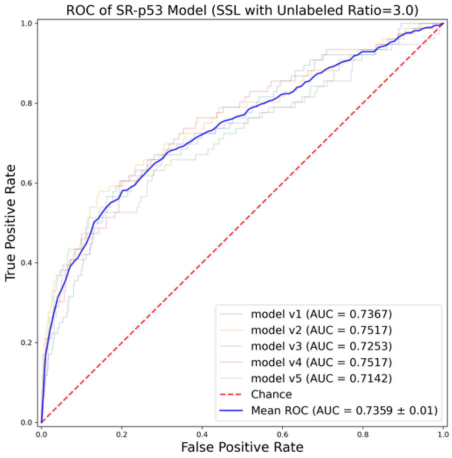

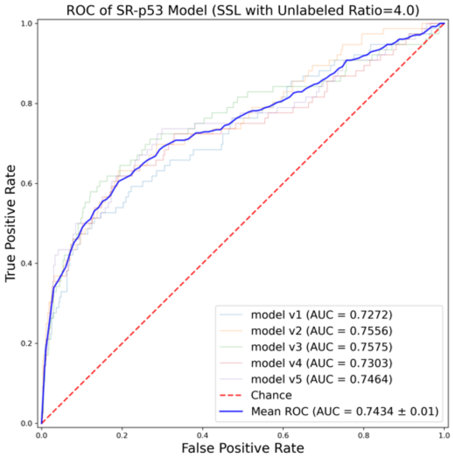

Figure S4. The distribution of SS_i in 12 similarity domains defined by different cutoff values C_s. The Z value of these cutoff values range from -2 to 3.5 with a step size of 0.5.

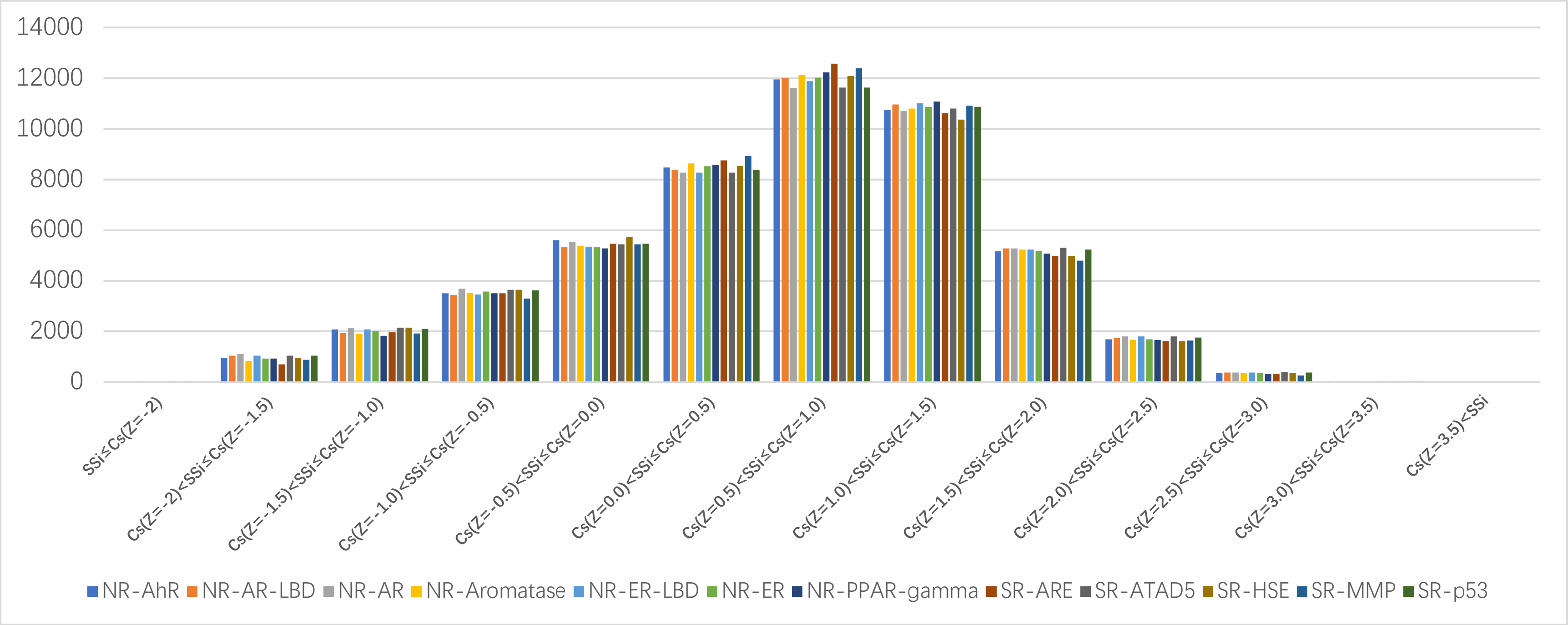

Supplement: Supplementary file 1 — Additional file 1: Table S1. Full test performances of conventional machine learning models on the 12prediction tasks in 5 repeated experiments. Table S2. Full test performances of SL-GCN on the 12 prediction tasks in 5 repeatedexperiments. Table S3. Full test performances of SSL-GCN on the 12 prediction tasks in 5 repeatedexperiments. The values in parentheses represent unlabeled data ratios (Ru). Table S4. Full test performance of the SSL-GCN models with different similarity levels of unlabeled subsets (close, normal, far) on the 12 prediction tasks in 5 repeated experiments. Figure S1. Details of the ROC curves of the conventional machine learning models in 5repeated experiments on the 12 prediction tasks. Figure S2. Details of the ROC curves of the SL-GCN models in 5 repeated experimentson the 12 prediction tasks. Figure S3. Details of the ROC curves of the SL-GCN models with different unlabeledratios (Ru) in 5 repeated experiments on the 12 prediction tasks. Figure S4. The distribution of SSi in 12 similarity domains defined by different cutoff values Cs. The Z value of these cutoff values range from − 2 to 3.5 with a step size of 0.5. [file 13321_2021_570_MOESM1_ESM.pdf]
